# Genetic diversity and structuring across the range of a widely distributed ladybird: focus on rear‐edge populations phenotypically divergent

**DOI:** 10.1002/ece3.2288

**Published:** 2016-07-13

**Authors:** Émilie Lecompte, Mohand‐Ameziane Bouanani, Alexandra Magro, Brigitte Crouau‐Roy

**Affiliations:** ^1^ Université Toulouse 3 UPS, UMR 5174 EDB (Laboratoire Évolution & Diversité Biologique)CNRS, ENFAF‐31062ToulouseFrance

**Keywords:** Bottleneck, *Coccinella*, local adaptation, Palearctic region, population genetics, rear‐edge populations

## Abstract

Population genetics and phenotypic structures are often predicted to vary along the geographic range of a species. This phenomenon would be accentuated for species with large range areas, with discontinuities and marginal populations. We herein compare the genetic patterns of central populations of *Coccinella septempunctata* L. with those of two phenotypically differentiated populations considered as rear‐edge populations and subspecies based on phenotype (Algeria and Japan). According to the central‐marginal model and expected characteristics of rear‐edge populations, we hypothesize that these rear‐edge populations have (1) a reduced genetic diversity, resulting from their relative isolation over long periods of time, (2) a higher population genetic differentiation, explained by low contemporary gene flow levels, and (3) a relationship between genetic diversity characteristics and phenotypes, due to historical isolation and/or local adaptation. Based on genotyping of 28 populations for 18 microsatellite markers, several levels of regional genetic diversity and differentiation are observed between and within populations, according to their localization: low within‐population genetic diversity and higher genetic differentiation of rear‐edge populations. The genetic structuring clearly dissociates the Algerian and Eastern Asia populations from the others. Geographical patterns of genetic diversity and differentiation support the hypothesis of the central‐marginal model. The pattern observed is in agreement with the phenotypic structure across species range. A clear genetic break between populations of Algeria, the Eastern Asia, and the remaining populations is a dominant feature of the data. Differential local adaptations, absence of gene flow between marginal and central populations, and/or incapacity to mate after colonization, have contributed to their distinct genotypic and phenotypic characteristics.

## Introduction

Processes such as genetic drift, gene flow, and natural selection impact the distribution of the genetic diversity and structuring across a species range. These processes may be strongly affected by both the species evolutionary history and its present demographic characteristics, such as population size, biotic and abiotic factors it might experience, or habitat fragmentation. A major paradigm explaining species distribution and population structure is the central‐marginal model. This model considers that core populations would exhibit increased abundance due to optimal conditions, whereas demographic parameters (reproduction and survivorship) should decline toward the edges (e.g., Brown 1984). Marginal populations are expected to be less genetically diverse and to present a potentially higher genetic differentiation, relative to central populations. Although these patterns are supported by numerous empirical studies, the decline in genetic diversity at range limits is not an ubiquitous trend (see Rajora et al. 1998; Gapare et al. 2005; Eckert et al. 2008; Neiva et al. 2012). This challenges the significance of such patterns at broad geographical scales (Sagarin and Gaines 2002; Vucetich and Waite 2003; Hampe and Petit 2005). Indeed, phylogeographic surveys show that past climate oscillations usually shaped population genetic diversity through range dynamics, with persistence of populations in refuge areas and recolonization events (see Bennett et al. 1991; Taberlet et al. 1998; Hewitt 2000, 2004; Petit et al. 2003). In that context, and particularly in the case of widespread species, two types of marginal populations must be distinguished – the leading‐ and the rear‐edge populations – modifying the expectations of the central‐marginal model according to the type of marginal population considered (see Hampe and Petit 2005; Guo 2012). The range expansion during postglacial events involves, in the Palearctic region, mostly populations from the colonization front (leading‐edge populations) located at the northern margin. A commonly observed consequence of rapid postglacial expansions is the decrease in genetic diversity both within and between populations inhabiting newly colonized areas, compared to those residing in persistently suitable habitats (Ibrahim et al. 1996; Hewitt 2000, 2004; Besold et al. 2008). However, we can observe an increased genetic diversity of northern populations (leading edges) due to the admixture of differentiated populations (Petit et al. 2003; Hewitt 2004). In contrast, the rear‐edge populations, in the periphery and/or isolated, have persisted in suitable habitat patches disjoint from the species’ continuous range. Only some of them have been the source of major postglacial recolonization (Bilton et al. 1998; Petit et al. 2003). These stable rear‐edge populations are often small in size and their long‐term isolation has resulted in reduced within‐population genetic diversity but in increased genetic differentiation, even between nearby populations. This leads to high and unique regional genetic diversity (Hampe et al. 2003; Petit et al. 2003; Hampe and Petit 2005; Guo 2012). Therefore, selection for local adaptation, rather than for vagility and generalism, is expected in these populations (Dynesius and Jansson 2000). Thus, in association with reduced gene flow, rear‐edge populations are more inclined to become genetically and phenotypically distinct and have a greater chance of speciation (Lesica and Allendorf 1995; Castric and Bernatchez 2003; Martin and Mckay 2004; Hardie and Hutchings 2010; Hoskin et al. 2011).

In this context, not only the population genetic structure of widespread species is predicted to vary along the geographic range (Vucetich and Waite 2003; Bridle and Vines 2007; Guo 2012), but also, in some cases, the phenotypic pattern (Hoskin et al. 2011). *Coccinella septempunctata* L. (Coleoptera: Coccinellidae), the seven‐spot ladybird, is an appropriate model to investigate the effect of location (core *vs* edges) on the genetic structure and diversity across a wide range. Indeed, being widely distributed across the Palearctic region, *C. septempunctata* experiences diverse biotic and abiotic environments. Its distribution expands from the Iberian Peninsula in the west to Japan in the east, to the Sahara in the south and the tundra in the north, but displays some discontinuities, especially in Siberia (Iablokoff‐Khnzorian 1982). *Coccinella septempunctata* is an ubiquitous species, feeding on a large number of aphid species (Hodek and Honek 1996), and therefore, it likely displays different life history strategies. Moreover, based on phenotypic characteristics and their location at margins, two populations have been recognized as distinct species, that is, the North African *C. algerica* Kovář (Kovář 1977) and the Japanese *C. brucki* Mulsant, later considered as a subspecies – *C. septempunctata brucki* (Dobzhansky and Sivertzev‐Dobzhansky, 1927). Marin et al. (2010) discussed the taxonomic status of *C. septempunctata* by combining the molecular data (ISSR) and the patterns of spots on the elytra, together with the assessment of potential barriers by crossbreeding. Although they found a high variation in the size of spots for the Japanese population, they showed a monophyly of all populations, without a clear genetic structuring along the range of the species, even for the marginal populations. ISSR provide large information to assess the genetic variability at perispecific level, but they might be inappropriate in an evolutionary history study (e.g., difficulty to identify alleles) and a further investigation, with codominant markers, is needed.

Due to its wide and discontinuous distribution and subsequent variation in ecological conditions, we hypothesize that *C. septempunctata* populations follow the hypothesis of “central *vs* marginal (leading and rear‐edge)” populations and therefore predict (1) marginal populations (rear edges, and maybe leading edges) less genetically diverse than the core ones, (2) a higher population structuring of the rear‐edge populations compared to the core, and potentially to the leading‐edge, populations, and (3) consistence between the phenotypic divergence observed for the rear‐edge Algerian and Japanese populations, and the genetic divergence due to historical isolation and/or local adaptation. Our objective is to characterize the patterns of genetic variability and structure in the rear‐edge populations by comparing populations across the entire distribution area. To uncover the factors involved in shaping the genetic structure over the range and to test whether its spatial structure is consistent with the central‐marginal model, we compared the genetic diversity of 28 sampled populations covering the native range, genotyping 407 individuals for 18 microsatellite markers developed from this species (Bouanani et al. 2015). More specifically, we focused on the genetic pattern of two phenotypically differentiated populations located at the rear edges: one in the south (Algeria) and the other in Eastern Asia (Japan).

## Materials and Methods

### Sample collection

**Figure 1:**
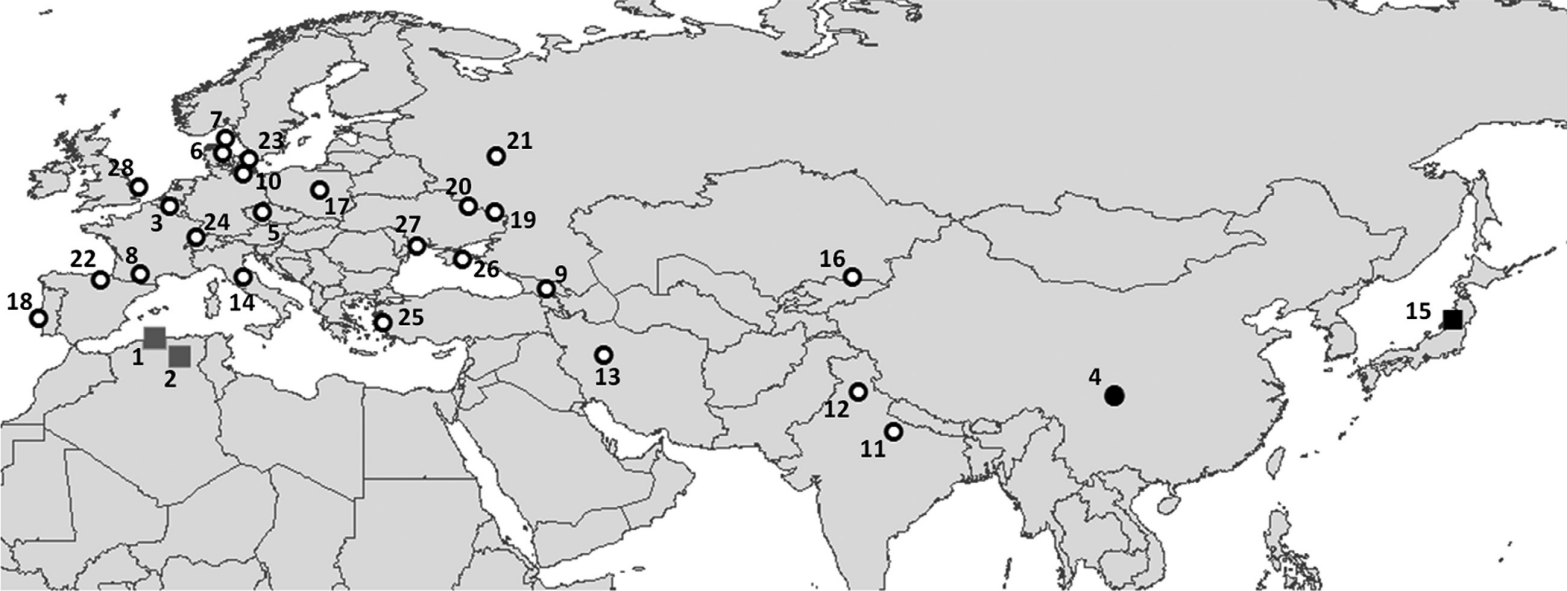
Locations of the 28 sampling sites for the seven‐spot ladybird (*Coccinella septempunctata*) in its native range. The colors of the dots refer to the clusters identified in the STRUCTURE analysis; the squares refer to populations considered a priori as rear‐edge populations: Algeria (1 and 2) and Japan (15). The northernmost populations were considered a priori as “leading” edge populations (Sweden, 23; Denmark, 6 and 7; Germany, 10; Russia, 21).

The individuals were sampled by several collectors in sites covering the entire native range of *C. septempunctata* species, from the core populations to the “leading” edge (northernmost limit: Sweden, Denmark, Germany, Russia) and to the “rear” edge at the southern and eastern limits, including a priori the populations from Algeria in the south and west and from Japan in the east (Table 1 and Fig. 1). The sample size being not identical in all the localities, we designed two datasets according to the analyses: one with the 28 populations (407 individuals), and a second including only populations with more than seven individuals (21 populations, 382 individuals). With the complete dataset, we did clustering analyses (STRUCTURE, discriminant analysis of principal components [DAPC]), while the second dataset was used for analyses at the population level (summary statistics: number of alleles, allelic richness [AR], expected and observed heterozygosities; *F*_ST_, between groups principal component analysis [PCA]).

**Table 1:** Characteristics of the sampling and summary statistics by population based on 18 microsatellites: sample size (*N*), mean number of alleles per locus (*A*), allelic richness (AR) (>6 individuals), mean expected (*H*_e_) and observed (*H*_o_) heterozygosities, with significant deviation from HWE indicated in bold

Sample locations		Collectors	Year	Latitude	Longitude	*N*	A	AR	*H*_e_	*H*_o_
1	Algeria, Alger	L. Saharahoui	2006	36.752887	3.042048	23	6.06	3.91	0.613	**0.481**
2	Algeria, Biskra	L. Saharahoui	2011	34.850000	5.733333	24	6.00	3.95	0.648	**0.540**
3	Belgium, Gembloux	L. Hautier	2007, 2011	50.564866	4.689044	32	8.33	4.62	0.675	**0.474**
4	China, Chengdu	S. Ponsard	2009	30.572305	104.066121	10	4.28	3.71	0.598	0.519
5	Czech Republic, Prague	A. Honek	2008	50.049339	14.439826	25	7.39	4.40	0.661	**0.552**
6	Denmark, Aarhus	S. Toft	2008	56.161433	10.204989	8	4.17	3.97	0.655	**0.476**
7	Denmark, Skagen	S. Toft	2011	57.724373	10.579031	24	7.72	4.52	0.660	**0.531**
8	France, Toulouse	F. Magné	2006	43.559280	1.472198	20	6.72	4.41	0.668	**0.538**
9	Georgia, Doesi	S. Barjadze	2007	41.934936	44.248357	2	2.50	–	–	–
10	Germany, Groß Lüsewitz	T. Thieme	2008	54.070964	12.338868	9	5.22	4.58	0.711	**0.584**
11	India, Lucknow city	Omkar	2007	26.851320	80.916803	15	5.89	4.31	0.639	**0.500**
12	India, Shimla	M. Kumari and D.C. Gautam	2007	31.104608	77.173418	22	7.00	4.37	0.665	**0.519**
13	Iran, Saveh	R. Kianpour and S. Moharramipour	2007	35.021774	50.357273	23	7.28	4.35	0.638	**0.533**
14	Italy, Perugia	J.‐L. Hemptinne	2010	43.110701	12.389172	13	6.06	4.46	0.664	**0.526**
15	Japan, Tsuruoka	J‐L. Hemptinne	2005	38.725528	139.825257	18	4.72	3.60	0.617	0.598
16	Kazakhstan, Kasskelen	S. Ponsard	2008	43.200002	76.619965	8	4.78	4.29	0.628	**0.430**
17	Poland, Tomianski	J.‐L. Hemptinne	2012	52.325502	20.435096	24	7.28	4.51	0.671	0.615
18	Portugal, Lisbon	J.C. Franco	2007	38.768632	−9.095858	25	6.94	4.37	0.657	**0.550**
19	Russia, Aydar	V. Aniskin	2008	50.048235	38.902285	3	2.39	–	–	–
20	Russia, Borisovka	V. Aniskin	2008	50.604162	36.015553	2	2.11	–	–	–
21	Russia, Orekhovo‐Zuyevo	V. Aniskin	2008	55.816477	38.983330	6	3.94	–	–	–
22	Spain, Vitoria‐Gasteiz	A. Magro	2010	42.850616	−2.707821	10	5.06	4.29	0.657	**0.428**
23	Sweden, Alnarp	E. Hatano	2009	55.649947	13.066672	13	6.11	4.52	0.679	**0.562**
24	Switzerland, Delemont	M. Kenis	2008	47.363511	7.357060	13	6.11	4.54	0.657	**0.467**
25	Turkey, Izmir	S. Ponsard	2012	38.418902	27.128782	2	2.28	–	–	–
26	Ukraine, Odessa	V. Aniskin	2008	46.482526	30.723308	3	3.17	–	–	–
27	Ukraine, Prymors'kyi	V. Aniskin	2008	45.112603	35.473476	7	4.61	–	0.700	**0.524**
28	United Kingdom, Norwich	A.F.G. Dixon	2008	52.630885	1.297357	23	7.28	4.57	0.673	**0.485**
	Total/Mean					407	5.407	4.30	0.656	0.520
							±1.80	±0.29	±0.03	±0.05

### DNA extraction, microsatellite amplification, and genotyping

Total genomic DNA was extracted from entire individual (minus elytra) using DNeasy Blood and tissue Kit (Qiagen, Valencia, CA) with PBS protocol according to the manufacturer's instructions. The 407 individuals were genotyped for 18 microsatellite markers, previously developed for the seven‐spot ladybird (Bouanani et al. 2015). The multiplex PCR was performed for each set of two to three loci in 10 μL of a mixture containing 10 ng of template DNA, 0.2 μmol/L of each primer, 5 μL of the Mix Qiagen Multiplex PCR kit and RNase‐free water. Each amplification, performed in an Eppendorf Mastercycler, consisted of an initial denaturation at 95°C for 15 min, 30 cycles of denaturation at 94°C for 30 s, hybridization at 60°C for 1 min 30 s and extension at 72°C for 1 min, and a final extension at 60°C for 30 min. For the populations from Algeria, China, and Japan, we optimized the amplification conditions for some loci (low annealing temperature and increased number of cycles) to obtain successful genotyping. The alleles were scored in the ABI 3130 XL at Genopole (Toulouse, France) using GeneMapper (version 3.7, Applied Biosystems Inc, Foster city, CA) for verifications and corrections. To verify the homology of some alleles, we sequenced, after cloning, the amplified product on both strands.

### Genetic diversity

The levels of genetic diversity (number of alleles per locus, Nei's unbiased expected heterozygosity, *H*_e_) of populations were computed with GENETIX v. 4.04 (Belkhir et al. 2004). To avoid biased estimates of genetic diversity due to sample size differences, we estimated the AR per locus and per sample, using the rarefaction approach in FSTAT v.2.9 software (Goudet 2001). When we excluded the populations with too small sample size (*N* < 7: Turkey, Georgia, two from Ukraine, and three from Russia), standard sample size consisted of the smallest population sample size with a complete genotype at all loci.

Tests for deviation from Hardy–Weinberg equilibrium (HWE) were conducted in GENEPOP (Rousset 2008), *P*‐values were obtained using a Markov chain of 100,000 steps. The linkage disequilibria among all locus‐pair combinations were computed using FSTAT v.2.9 (Goudet 2001) and the corresponding *P*‐values were adjusted using Fisher's exact test with 10,000 permutations. We tested for large allele dropout, null alleles, and stuttering that could explain deviations from HWE using MICRO‐CHECKER v2.2.3 (Van Oosterhout et al. 2004). We estimated the null allele frequencies for each locus and population using FreeNA (Chapuis and Estoup 2007). In each site, the departure from random mating (inbreeding coefficient: *F*_IS_) was tested using FSTAT (Goudet 2001) by permuting alleles (10,000 permutations) among individuals within populations.

The AR and gene diversity (*H*_s_) were compared between several groups of populations using FSTAT (Goudet 2001) with 10,000 permutations. Populations were clustered following the population differentiation estimated from *F*_ST_ values, and the outcomes of the PCA and the clustering analyses.

### Population genetic structure and differentiation

To test for population differentiation, pairwise *F*_ST_ using FSTAT (Goudet 2001) were calculated, the significance of differentiation was tested permuting genotypes (10,000 permutations) among localities, and the corresponding *P*‐values were adjusted for multiple comparisons with a Bonferroni procedure (*α *= 0.05).

We estimated isolation by distance (IBD), analyzing correlation between the pairwise linearized genetic differentiation of populations (*F*_ST_/(1 − *F*_ST_)) and log‐transformed geographic distances calculated as the linear distance between sampling sites in km. Mantel tests were conducted, with the ade4 package (Chessel et al. 2004) for R 3.2.2 (R Development Core Team 2015) with 10,000 permutations, on the 21‐populations dataset, with and without the populations from Algeria, China, and Japan.

The overall genetic structure was analyzed using PCA based on allele frequencies for each population. The between‐group PCA was performed using the adegenet package (Jombart 2008) for R 3.2.2 (R Development Core Team 2015); the missing data were replaced with the mean allele frequency. Hierarchical analysis of molecular variance was computed using Arlequin v3.5 (Excoffier et al. 2005) and the significance of the genetic structure tested using 10,000 permutations. Variance components were extracted for three hierarchical levels (1) among individuals within localities, (2) among localities within genetic groups, and (3) among genetic groups. Genetic groups were partitioned following the population differentiation estimated from *F*_ST_ values, and the outcomes of the PCA and the clustering analyses.

### Genetic clustering of individuals

Multilocus genotypes were used to infer clusters of individuals representing different gene pools. A Bayesian method (with Markov chain Monte Carlo [MCMC], estimation) was used based on the data from all populations, as implemented in STRUCTURE version 2.2 (Pritchard et al. 2000), without using prior population information or spatial data. We conducted the STRUCTURE analysis over the entire dataset (407 individuals from all populations), with an admixture model with correlated allele frequencies and burn‐in period of 50,000 and 1,000,000 MCMC generations, respectively. Ten independent runs for each number of clusters (*K*) were conducted, *K* varying from 1 to 15. The optimum number of clusters was determined according to the delta *K* method (Evanno et al. 2005). The ten runs of the selected *K* were then aligned together in a single run using CLUMPP version 1.1.2 (Jakobsson and Rosenberg 2007). The cluster graphs were produced from the CLUMPP output files using DISTRUCT version 1.1 (Rosenberg 2004).

We used the DAPC, a model‐free multivariate method, to identify genetic clusters when prior grouping information is lacking (Jombart et al. 2010). This is a clustering analysis that first performs a PCA, then a discriminant analysis on the PC scores. We performed DAPC and graphically displayed our results using adegenet (Jombart 2008) and ade4 packages (Chessel et al. 2004) for R 3.2.2 (R Development Core Team 2015). The inference of the most likely number of clusters was based on the Bayesian information criterion.

### Inference of past demographic processes

BOTTLENECK was used to test for recent population bottlenecks and expansions in the populations containing more than 10 individuals, as recommended by authors (Piry et al. 1999). We tested whether the expected heterozygosity (*H*_e_) is significantly higher or below the heterozygosity predicted at mutation – drift equilibrium (*H*_eq_) on the basis of the observed number of alleles (*N* < 20), that is, likely to arise from a population size reduction or expansion, respectively (Cornuet and Luikart 1996). The significance of the analyses was assessed with one‐tailed Wilcoxon's signed‐rank tests based on 10,000 replications. The stepwise mutation model (SMM; Ohta and Kimura 1973), which is reliable for microsatellite data, was used (Luikart and Cornuet 1998), as well as the two‐phase model of mutation (TPM) with various percentages of multistep changes (5, 20, 30, 50, 70%) and a variance of 12 among multiple steps, because few microsatellites follow the strict SMM (Di Rienzo et al. 1994). Moreover, to test for biases due to the effect of potential null alleles, we realized the analyses (1) discarding the five loci with the highest estimated NA frequency (>19%) and (2) with the six loci where a very low frequency of NA were estimated (mean across populations <3%, Table S2).

## Results

### Patterns of genetic diversity

A total of 407 individuals from 28 populations, covering the native range of the species, were genotyped and analyzed at 18 polymorphic microsatellite loci. A total of 300 alleles were detected in the complete dataset, with in average 16.5 (SD = 8.4) alleles/locus. No significant linkage disequilibrium was detected between any pair of loci across all populations (*P* > 0.05 after Bonferroni correction). For populations with more than seven individuals (21 populations), the average number of alleles per locus ranged from 4.17 to 8.33, with a mean of 6.14 (SD = 1.2). The average expected heterozygosity (*H*_e_) was 0.656 (SD = 0.03) ranging from 0.598 (China) to 0.711 (Germany), and the mean observed heterozygosity (*H*_o_) was 0.52 (SD = 0.05) varying from 0.428 (Spain) to 0.615 (Poland). Detailed data for the genetic parameters are given in Table 1.

Globally, the populations showed a significant deviation from the HWE (Tables 1 and S1). This heterozygote deficiency suggested that significant null allele frequencies exist. We estimated significant null allele frequencies for all loci, but not consistently for the same locus across all the populations. This suggested no systematic biases in PCR amplification, with the exception of three loci (di154, di261, and di310), for which a high estimated average frequency of null alleles (>19%, Table S2) was observed in most of the populations.

The mean AR, standardized for seven individuals per population, was 4.30 (SD = 0.29), ranging from 3.60 (Japan, *N* = 15) to 4.62 (Belgium, *N* = 32); the mean expected heterozygosity (*H*_e_) was 0.656 (SD = 0.03) ranging from 0.598 (China, *N* = 10) to 0.711 (Germany, *N* = 9; Table 1). The populations showing the lower genetic diversity were rear‐edge populations: the two Algerian populations (AR = 3.91 and 3.95; *H*_e_ = 0.613 and 0.648; *N* = 23–24), Japan (AR = 3.60; *H*_e_ = 0.617; *N* = 15), and China (AR = 3.71; *H*_e_ = 0.598; *N* = 10). Populations from northern limits were highly diversified (e.g., Denmark, Skagen: *H*_e_ = 6.660; AR = 4.15 or Sweden: *H*_e_ = 6.679; AR = 4.17), as well as core populations (e.g., Italy: *H*_e_ = 6.664; AR = 4.11 or Czech Republic: *H*_e_ = 6.661; AR = 4.05; Table 1). We compared the genetic diversity of the cluster including rear‐edge populations (Algeria and Japan) plus China (according to the structuring analyses, see below) to the other populations. This cluster had significantly lower genetic diversity than the other ones (AR = 3.79 versus AR = 4.41, *P* = 0.0001 and *H*_s_ = 0.63 vs. *H*_s_ = 0.67, *P* = 0.0012). Among the other populations, no significant difference (*P* > 0.50) was observed between the leading‐edge populations (the northern ones: Germany, Denmark, and Sweden, AR = 4.39, *H*_s_ = 0.67) and populations from potential refugia that could have participated to the colonization of northern areas (the southern ones: Italy, Spain, Portugal, AR = 0.47, *H*_s_ = 0.66).

### Patterns of genetic structure and differentiation

The hierarchical analyses of molecular variance revealed significant partitioning of genetic variation among groups of populations and among populations within groups (Table 2). We observed a significant global *F*_ST_ (*P* < 0.001) among the 21 sampling sites, explaining 6.38% of the variation. A hierarchical structure was observed between the three groups (Algeria, Eastern Asia (China and Japan), and all the other populations, *F*_CT_ = 0.116, *P* < 0.001). Such partitioning explained 11.6% of the genetic variance and 86.5% was within populations (see Table 2).

**Table 2:** Analysis of molecular variance in 21 populations of *Coccinella septempunctata* based on 18 polymorphic microsatellites. Three groups were defined according to the results from the PCA and STRUCTURE analyses: Algeria, Eastern Asia (Japan and China), and all other populations

Source of variation	Sum of squares components	Variance	% Variation	*F*‐statistics	*P*‐value
Among groups	217.9	0.769	11.57	*F*_CT_ = 0.116	<0.001
Among populations within groups	192.1	0.126	1.90	*F*_SC_ = 0.021	<0.001
Within populations	4349.2	5.753	86.54	*F*_ST_ = 0.135	<0.001
Total	4759.2	6.648			

Global *F*_ST_ among localities without hierarchy is 0.064, *P* < 0.001.

Global analyses of genetic differentiation revealed highly variable *F*_ST_ values, comprised between 0 and 0.24, with a high and statistically significant differentiation between each of the four populations (Algeria [×2], China, and Japan) and all the others. More specifically, the highest genetic differentiations were observed between the Algerian (no significant differentiation between the two populations sampled) and the Japanese or Chinese populations (*F*_ST_ = 0.17–0.24, *P* < 0.001), between Japanese and Chinese populations (*F*_ST_ = 0.13, *P* < 0.001), and between Algerian, Japanese, or Chinese populations with all the other populations (*F*_ST_ = 0.09–0.18, *P* < 0.001). With the exception of the pairwise comparisons including the Algerian, Chinese, or Japanese populations, pairwise *F*_ST_‐estimates were generally low, and many of them were not statistically different from zero, except for some pairwise comparisons, including mainly comparisons with populations of India and Iran (17 on 153 pairwise *F*_ST_ values, *F*_ST_ = 0.03–0.04, *P* < 0.05).

Individuals from Algeria, Japan, and China, unlike most of the populations, showed some particularities in their genotyping supporting their genetic differentiation. First, in the standard conditions, the success of genotyping for two markers was lower than for the other populations (tetra118 for Algerian populations and tetra112 and tetra118 for the Japanese and Chinese populations), suggesting substitutions and/or indels in the adjacent region of the repeats where the primers were designed. Second, we detected a number of alleles in Japanese samples either with a high PCR product size or a very low size, suggesting a high number of repeats or no repeat at all (related to the observed size and the number of repeats in the reference sequence). These private alleles or shared for some of them with only the Chinese sample were sequenced and revealed deletions or insertions in the adjacent region of the repeat. For example, the allele “191” of the di208 locus, at high frequency in the Japanese population (freq = 0.39; eight homozygote individuals), revealed four AG repeats and a deletion of 14 bases; the allele “267” of the di282 locus, with a frequency of 0.472 in Japan, showed five AC repeats plus a deletion of 15 bases (Fig. S1).

**Figure 2:**
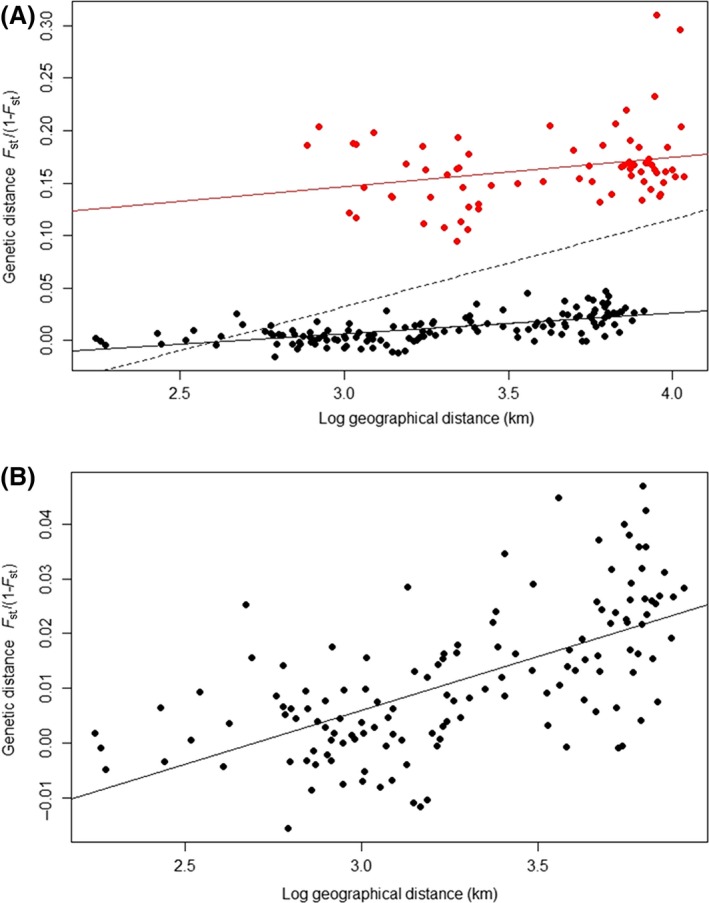
Isolation by distance (IBD) in *Coccinella septempunctata*: regression plots of the genetic distance between populations (*F*_ST_/(1 −* F*_ST_)) against the log of Euclidean distance. (A) All population pairs, with the regression as dotted line. Dots indicate pairwise population comparisons: in red, between Algeria, China or Japan, and all the other populations, and in black, between the 24 remaining populations, with their independent regression lines. (B) Pairs of populations with the exception of populations from Algeria, China, and Japan.

The signal of IBD over all data is complex, with two discrete clusters of points (Fig. 2A; Mantel test *P* = 0.0036; *r* = 0.47): a cluster of extremely differentiated populations encompassing populations from Algeria, China, and Japan and a cluster of poorly differentiated populations (all the remaining populations, *N* = 24). This pattern is not due to a stepping‐stone dispersal consistent with IBD, but to the high differentiation of the populations from Algeria, China, and Japan compared to the others. When we excluded these four populations, we observed a significant pattern of IBD (Fig. 2B; *P* < 0.001) with coefficient of determination higher (*r* = 0.63) than for the complete dataset.

**Figure 3:**
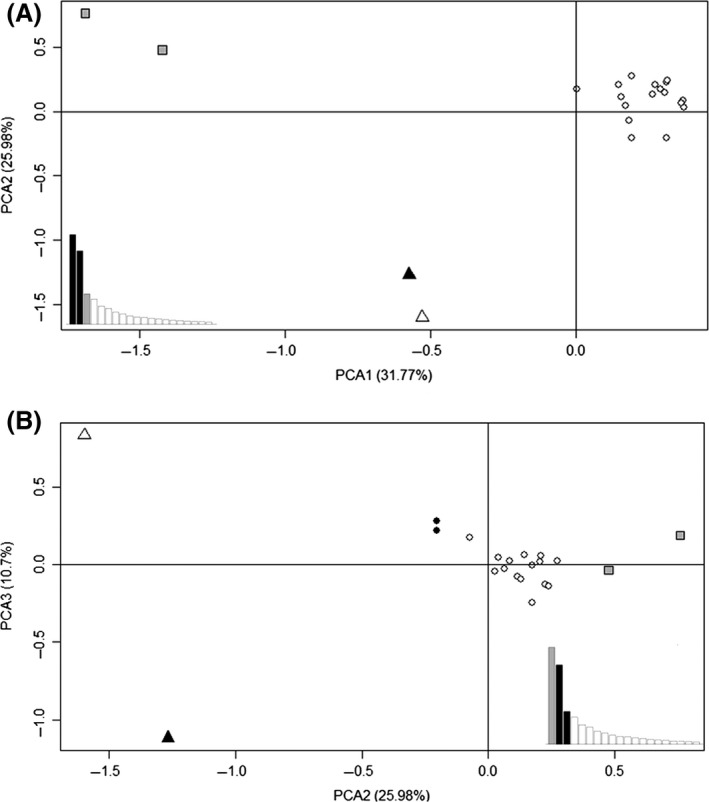
Principal component analysis (PCA) based on allele frequencies of populations of 18 microsatellite loci. (A) PCA1 separates rear‐edge populations (Algeria in square gray, East Asia as triangle: Japan in black, China in white), whereas PC2 separates populations from Eastern Asia (Japan and China) from the others (white circles). PC1 and PC2 explain 37.77 and 25.98% of the variance, respectively. Eigenvalues for the components are indicated with the first two components shown in black. (B) PC2 dissociates populations from Eastern Asia (Japan in black square and China in white), Algeria (gray), and India (black point) from the others (white). PCA3 separates the populations from Japan and China. PCA2 and PCA3 explain 25.98 and 10.7% of the variance, respectively. Eigenvalues for the components are indicated with the components PCA2 and PCA3 shown in black.

The PCA analysis also showed a high level of genetic differentiation between the populations from Algeria (southern edge), Japan and China (Eastern Asia), and each of the other populations (Fig. 3). The first axis, explaining 32% of the variance, separated the rear‐edge (Algeria and Japan) and Chinese populations from the others; the second axis (26%) separated the Chinese and Japanese populations from the others (Fig. 3A). The third axis (11% of the variance) dissociated the China from the Japan populations (Fig. 3B).

**Figure 4:**
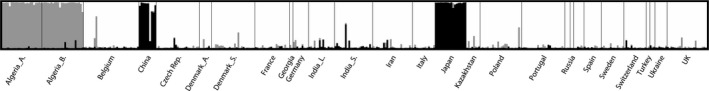
Results of the Bayesian structure analyses of *Coccinella septempunctata* populations across Palearctic, calculated with the program STRUCTURE. Bar plot shows proportions of individual multilocus genotypes assigned to each of the most probable clusters (*K* = 3), illustrated by the different colors. The vertical lines separate the 28 sampled populations (in alphabetical order) listed in Table 1.

**Figure 5:**
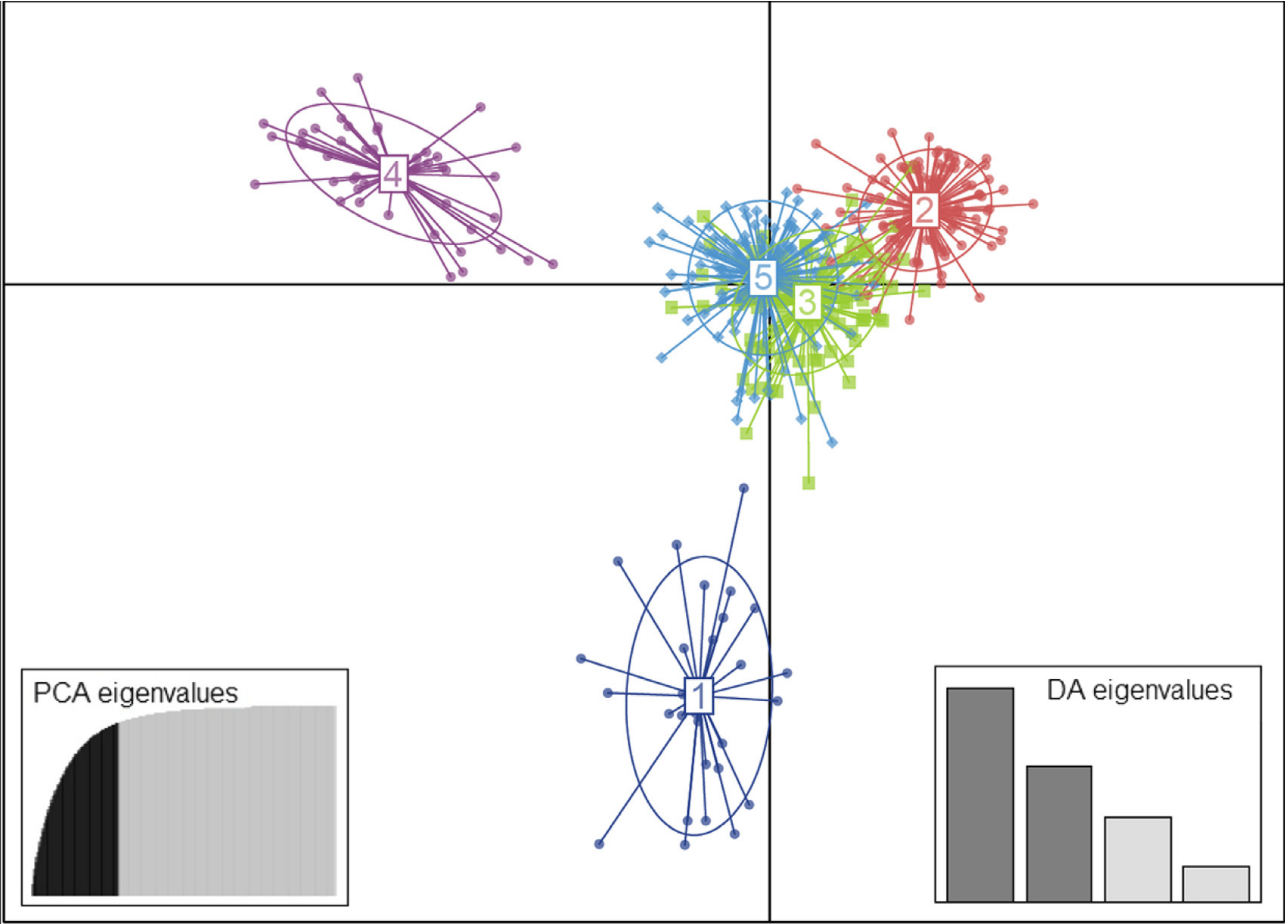
Scatter plot of the discriminant analysis of principal components (DAPC). Each of the five clusters is depicted by distinct color inside their 95% inertia ellipses and dots represent individuals. The axes represent the first two discriminant functions, respectively. The plots of eigenvalues show the amount of genetic information retained by the PCA (on the left) and the discriminant function (DA, on the right).

The individual‐based clustering approaches (STRUCTURE, DAPC) provided evidence of genetic substructuring among populations. The STRUCTURE analysis revealed an optimal number of genetic clusters of three (*K* = 3), discriminating Algerian populations from Eastern Asia populations (China and Japan) and from a cluster comprising all the 24 other localities (Fig. 4). The inferred population structure showed that 98% of the individuals have a membership coefficient to one of the clusters higher than 90%. We identified no substructuring within the cluster of 24 populations, identified at *K* = 3 or at a higher number of genetic clusters (Fig. S1). Only few individuals were admixed and this result suggested little gene flow, ancient or recent, between clusters (Fig. 4). When we analyzed only the samples from the 24‐population cluster (without Algerian, Japanese, and Chinese samples), no particular structure was revealed. The first split, separating Algerian and Eastern Asian samples from all others, appeared from *K* = 2 (Fig. S2). The DAPC inferred the optimal number of genetic clusters as five (*K* = 5). The first principal component axis (eigenvalue = 894.8) separated one cluster (individuals from Algeria) from all the others (Fig. 5); the second axis (eigenvalue = 566) separated a cluster (populations from China and Japan), from the others (Fig. 5). The distributions of the three other genetic clusters (clusters 2, 3, and 5), which comprise the 24 populations clustered in STRUCTURE, overlapped. This suggested a low degree of genetic differentiation among these populations. Each of these populations, but three (with a low sample size, *N* < 3), was attributed to the three clusters (Fig. S3). The relative frequencies of the components of the three clusters in the populations showed no geographical pattern (Fig. S3).

### Inference of past demographic processes

The Wilcoxon's test, for the null hypothesis of no significant heterozygosity excess or deficit across loci, showed a significant deficit of heterozygosity for most of populations, which may reflect a recent demographic expansion. However, these results varied according to the mutation model, the SMM model (or high proportion of stepwise mutations in the TPM model) being more prone to yield significant signatures of expansion (Table S3). Conversely, a signature of bottleneck was detected in Japan and China under the TPM model of mutation, with a small proportion (5–30%) of stepwise mutations in the TPM model (Table S3). Moreover, when loci, with a significant NA frequency, were excluded, most of the results were similar, except for China (Table S3).

## Discussion

In widespread species, the selective and demographic histories can be intricate. Moreover, these species are not always continuously distributed and may show isolated populations particularly in the range periphery. Thus, not only the population genetic structure is predicted to vary along the geographic range of the species (Vucetich and Waite 2003; Bridle and Vines 2007; Guo 2012) but also, in some cases, the degree of phenotypic differentiation between populations (Hoskin et al. 2011). Due to the different ecological conditions, found along its wide and discontinuous distribution, and to the phenotypic variations observed across its range, the patterns of genetic variability and structuring of the *C. septempunctata* populations could be consistent with the central‐marginal model (Petit et al. 2003; Martin and Mckay 2004; Hampe and Petit 2005; Eckert et al. 2008).

### Low genetic diversity and high population differentiation in rear edges

Distinct levels of regional genetic diversity and differentiation were identified using nuclear polymorphic markers. More specifically, the rear‐edge populations from Algeria (southern limit) and Eastern Asia (Japan and China) show particularities, both in terms of differentiation and genetic diversity. Taken together, our results suggest the presence of three main clusters with the populations from Algeria and Eastern Asia (Japan and China) highly genetically separated from the rest of the populations sampled. Indeed, the level of genetic differentiation between the core populations and the southern (Algeria) and eastern (China + Japan) edge populations is significantly higher than between the core populations. This suggests a low gene flow, either contemporary or historical, between these populations. Our results differ from the study based on polymorphic nuclear markers (ISSR), where no geographic pattern was identified (Marin et al. 2010). However, as ISSR polymorphisms are not able to identify homologous alleles, they might be less reliable than microsatellite data to infer the evolutionary history and genetic structure at this scale. The rear‐edge populations, together with China, have significantly lower genetic diversity than the other populations (core and northern ones), where the high genetic diversity is consistent with the large effective population size expected in central populations. Evidence for a recent bottleneck was detected only in Chinese and Japanese populations, also supported by their low levels of genetic diversity. However, subsequent demographic changes may erase the signatures of bottleneck, even if an extreme population decline occurred (e.g., Luikart et al. 1998; Busch et al. 2007; Mc Eachern et al. 2011). Actually, the signatures of bottleneck likely depend on the population growth rate and admixtures (Mc Eachern et al. 2011). In this study, a signature of expansion was detected in almost all the populations, including the marginal populations from Algeria (Table S3). The bottleneck or expansion signals observed may have been biased by the presence of null alleles. However, the null alleles would be a minor source of error in the test because (1) the observed heterozygosity is not used but only the comparison of two types of expected heterozygosity (*H*_e_ and *H*_eq_), (2) all the populations would potentially be impacted by their presence, and we identified a signature of either expansion or bottleneck according to the populations, and (3) the patterns were overall similar with or without loci with significant frequency of NA.

Interestingly, despite the large sampled area, no obvious pattern emerges either for genetic diversity or differentiation within and between the northern and core populations. Nevertheless, significant IBD was detected within the apparent well‐mixed core and northern pool of populations, suggesting high level of dispersal and extensive admixture preventing differentiation. These findings were also supported by the signature of population expansion identified in almost all populations. Our results, a priori not consistent with the central‐marginal model, are coherent with the presence, in a large part of the range, of postglacial recolonization, which have induced the expansion and the admixture of populations previously isolated, leading to high genetic diversity and low differentiation (Petit et al. 2003; Hewitt 2004; Hampe and Petit 2005; Guo 2012).

### Different evolutionary histories on southern and eastern edges

The low within‐population diversity and the high differentiation recorded for the marginal populations at the southern (Algeria) and eastern edges (Japan, associated with China) are consistent with the features expected for rear‐edge populations (see Hampe and Petit 2005). Moreover, these rear‐edge populations, characterized by distinct phenotype patterns and environmental conditions, also differ by the geographical barriers isolating them from the core of the distribution. Consequently, we expected different evolutionary histories between populations from eastern and southern rear edges.

#### Eastern edge: Japanese and Chinese populations

We confirm the distinctiveness of the Japanese population, recognized as a different subspecies, based on the elytral spots and the persistence of divergent mtDNA haplotypes (Marin et al. 2010; Kajita et al. 2012). In addition, our results clearly indicate, for the first time based on molecular data, that populations from China and Japan are closely related. Interestingly, the differentiation observed is supported by numerous private and divergent alleles in Japan or in the group “Eastern Asia”, which differ not only by variations of repeat number but also by indels in the adjacent region of the repeats. These insertion/deletions, good markers to infer evolutionary relationships (Grimaldi and Crouau‐Roy 1997), reveal a strong signature of the differentiation of the Eastern Asian populations. These results strongly suggest that the Japanese population was not completely isolated from the continent. However, the significant high level of genetic differentiation observed between China and Japan (*F*_ST_ = 0.13, PCA) suggests limitation of gene flow, likely due to ancient isolation. Indeed, during the Last Glacial Maximum (c.a. 18 ka and possibly earlier cold periods), a land bridge across the East China Sea used to connect the currently isolated region of Japan (see Harrison et al. 2001), allowing intermittent gene flows between the Asian mainland and Japan, with periodic secondary contacts. The cluster “Eastern Asia” harbors genetic specificities, revealed by both microsatellite private alleles and by mitochondrial haplotypes, at least for Japan (Marin et al. 2010). The absence of these private alleles and mitochondrial lineages in the core populations suggests that Eastern Asian populations did not contribute to the postglacial colonization of previously glaciated areas. The population from China could have been isolated from the rest of the Asian continent. The distribution is discontinuous in Asia, the species being absent from Siberia and therefore isolating Japan and China but also populations of the Pacific coast of Siberia from the rest of the range area (Iablokoff‐Khnzorian 1982). Moreover, while populations from India are geographically relatively closed to China, we identified them as highly divergent from Chinese populations, likely due to the presence of the Himalaya Mountains, barriers for the dispersion of ladybirds. The bottleneck signatures observed in China and Japan populations, associated with the high genetic differentiation and a phenotypic divergence, suggest that these two populations follow independent evolutionary trajectories from the core populations and could have a greater chance of speciation (Martin and Mckay 2004; Hoskin et al. 2011). Indeed, although genetic divergence of allopatric populations does not define a speciation event, it is part of the factors leading to a possible speciation (Slatkin 1987; Turelli et al. 2001). Their evolutionary independence would increase the likelihood of a divergence of traits important in reproductive isolation and speciation (Martin and Mckay 2004).

#### Southern edge: Algeria

The North African populations were considered as a distinct species, *C. algerica* (Kova 1977), based mainly on subtle differences in the shape of the elytra and pronotum, the elytral spots and the male tegmen. Later, morphometric characteristics of elytral spots did not allow discriminating these populations from the others, despite marginally larger spots (Marin et al. 2010). In addition, previous molecular analyses show either an absence or a low genetic differentiation from the other populations, suggesting that the Algerian population could not be recognized as a distinct species (Marin et al. 2010). Conversely, our results clearly show a high level of genetic differentiation of Algerian populations compared to all the other populations, even with the nearest Portuguese and Spanish populations (*F*_ST_ = 0.12–0.17, *P* < 0.001). This suggests either absence or limited gene flow between North Africa and Europe. However, some individuals attributed to *C. algerica* have been found in sympatry with the typical form in Gibraltar (Bensusan et al. 2006). Although dispersion and associated gene flow between North Africa and Europe is possible across Gibraltar's strait, we do not know whether *C. algerica* presence in Gibraltar is due to their large dispersal capacity or to human assistance. Because of the level of differentiation observed, the restriction to gene flow is likely related to the incapacity or low possibility to mate in the field between Algerian and European samples, potentially due to local adaptation, rather than to the presence of geographical barrier. Core populations are expected to experience a much larger range of environments promoting a stabilizing selection, while populations at the margins are expected to experience fluctuating environmental conditions and then potentially divergent selection (Dynesius and Jansson 2000). Organisms in fluctuating environments must constantly adapt to these changes by responding appropriately, for example, by switching phenotype or behavior. Moreover, the long‐term persistence in quaternary refugia for the North Africa populations (Husemann et al. 2014), associated with a small population size, could explain their low genetic diversity as well as their local adaptation, which could promote phenotypic divergence and potentially speciation (Schluter 2001; Hoskin et al. 2011). Weak differential selection (i.e., subtle differences in environment) can dramatically increase the rate of divergence when gene flow is absent or limited (e.g., Rice and Hostert 1993; Gavrilets 2003). Thus, field mating between Algerian and European populations may be either impossible or less efficient (with reduced offspring fitness). For example, outbreeding depression has been shown in the mating of sea grass populations, even across short distances (Billingham et al. 2007). The differential local adaptation combined with restricted gene flow between Algerian and central populations may have contributed to their differentiation, both genetically and phenotypically. Such hypothesis may be supported by phenology differences (voltinism and timing of resumption of activity after diapause) between populations (Hodek and Honek 1996; Bensusan et al. 2006). It would be informative to experimentally crossbreed the Algerian and European individuals, as it was realized between Japanese and European samples (Marin et al. 2010). However, artificial crosses used to delimit species have been questioned because they may bypass some premating barriers to hybridization such as differences of phenology or social structure. In addition, a fine sampling of *C. septempunctata* populations in North Africa, Spain, and Portugal could allow detecting ongoing gene flow in the field, allowing clarifying the status of the North African populations.

## Conclusions

This study investigates the spatial genetic diversity of the *C. septempunctata* in its range area. Our results reveal a high genetic structuring pattern, not evidenced previously by either ISSR loci or mitochondrial haplotypes, with the Algerian and Japanese rear‐edge populations, highly differentiated, that is consistent with their morphological distinctiveness. We also identify a close proximity between the Chinese and Japanese populations (eastern edge). A further phylogeographical study, confronting sequences from both nuclear and mitochondrial markers, could provide a better knowledge of the patterns of genetic structure and diversity in the native range and be useful to evaluate the historical influences (impact of glaciations on genetic diversity and structuring) on the contemporary genetic patterns.

## Conflict of Interest

None declared.

## Supporting information


**Figure S1.** Plots of population allele frequencies per locus represented by dots of varying size. The two populations from Algeria are clustered.

**Figure S2.** Results of the Bayesian structure analyses of *Coccinella septempunctata* populations across Palearctic, calculated with the program STRUCTURE.

**Figure S3.** DAPC assignment: posterior probability of assignment of each individual to the five DAPC clusters.

**Table S1.** *F*_IS_ values, per population and per locus, and across all loci with the significant values indicated in bold.

**Table S2**. Null allele frequencies estimated per locus and per population. “–” means that null alleles were not encountered.

**Table S3**. Genetic signatures of demographic changes in *Coccinella septempunctata* identified with BOTTLENECK, significant deficiency of heterozygotes are indicated as “expansion” and excess as “bottleneck”.
