# Supplementary material for: Genetic diversity and structuring across the range of a widely distributed ladybird: focus on rear‐edge populations phenotypically divergent
Source: Ecol Evol. 2016 Jul 13;6(15):5517–29. doi: 10.1002/ece3.2288 (PMC4984522; doi:10.1002/ece3.2288)
Supplement: Supplementary file 1 — Figure S1. Plots of population allele frequencies per locus represented by dots of varying size. The two populations from Algeria are clustered. [file ECE3-6-5517-s001.pdf]

Locus di130 – 12 alleles

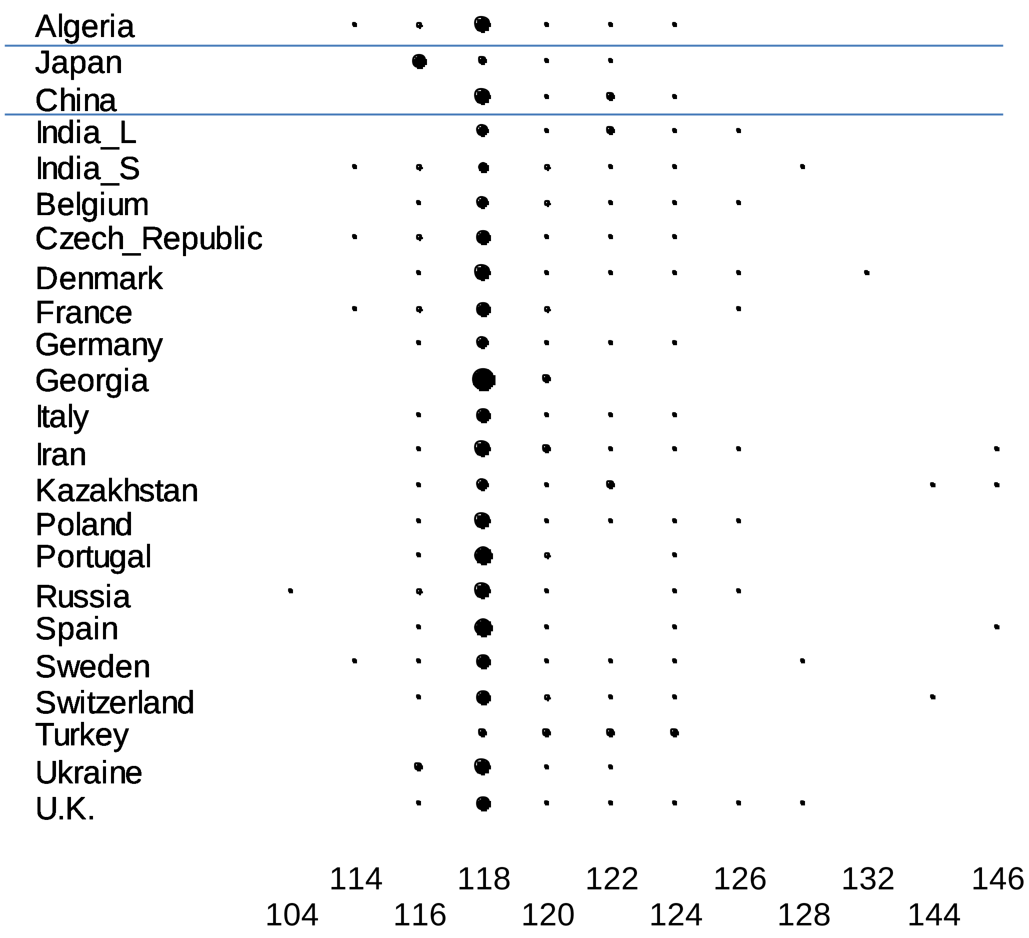

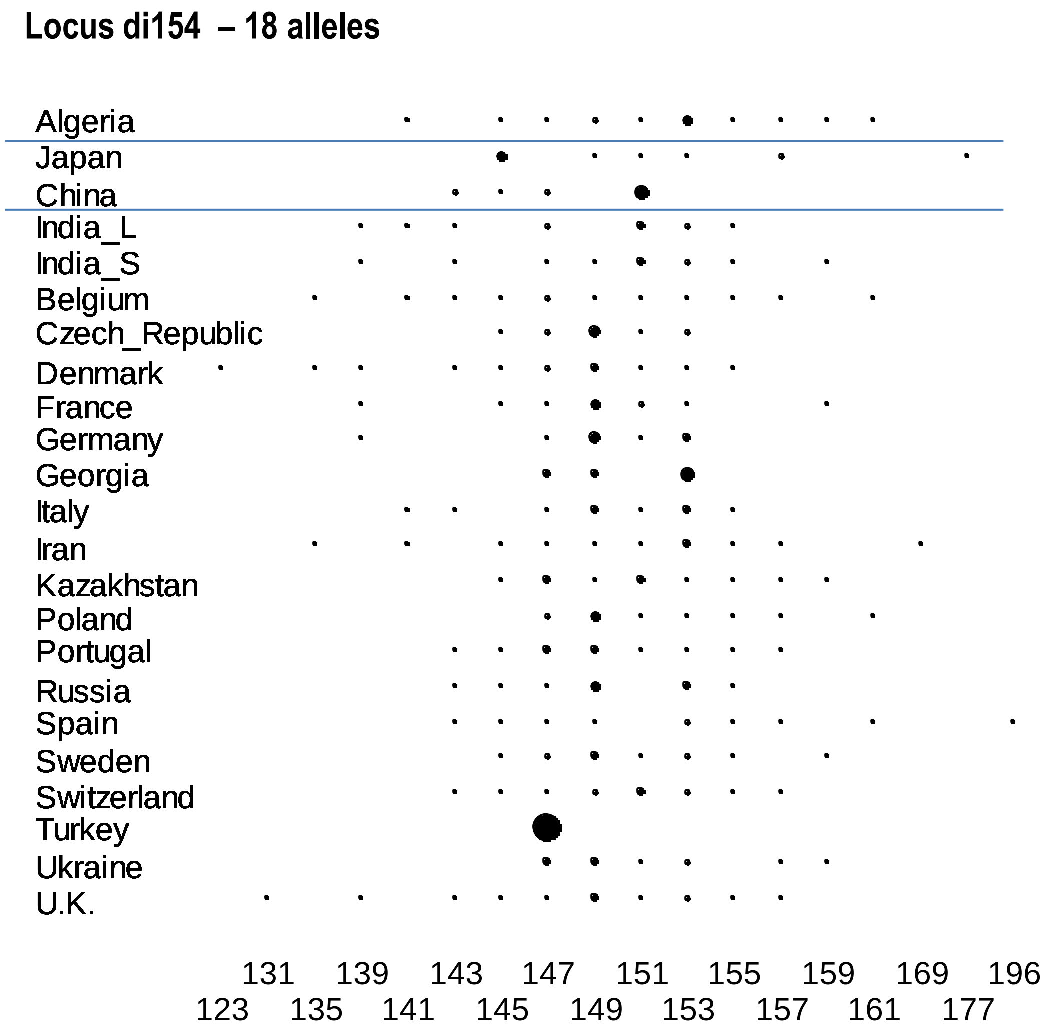

Locus di154 – 18 alleles
Algeria
Japan
China
India_L
India_S
Belgium
Czech_Republic
Denmark
France
Germany
Georgia
Italy
Iran
Kazakhstan
Poland
Portugal
Russia
Spain
Sweden
Switzerland
Turkey
Ukraine
U.K.
131 139 143 147 151 155 159 169 196
123 135 141 145 149 153 157 161 177

Locus di155 – 16 alleles

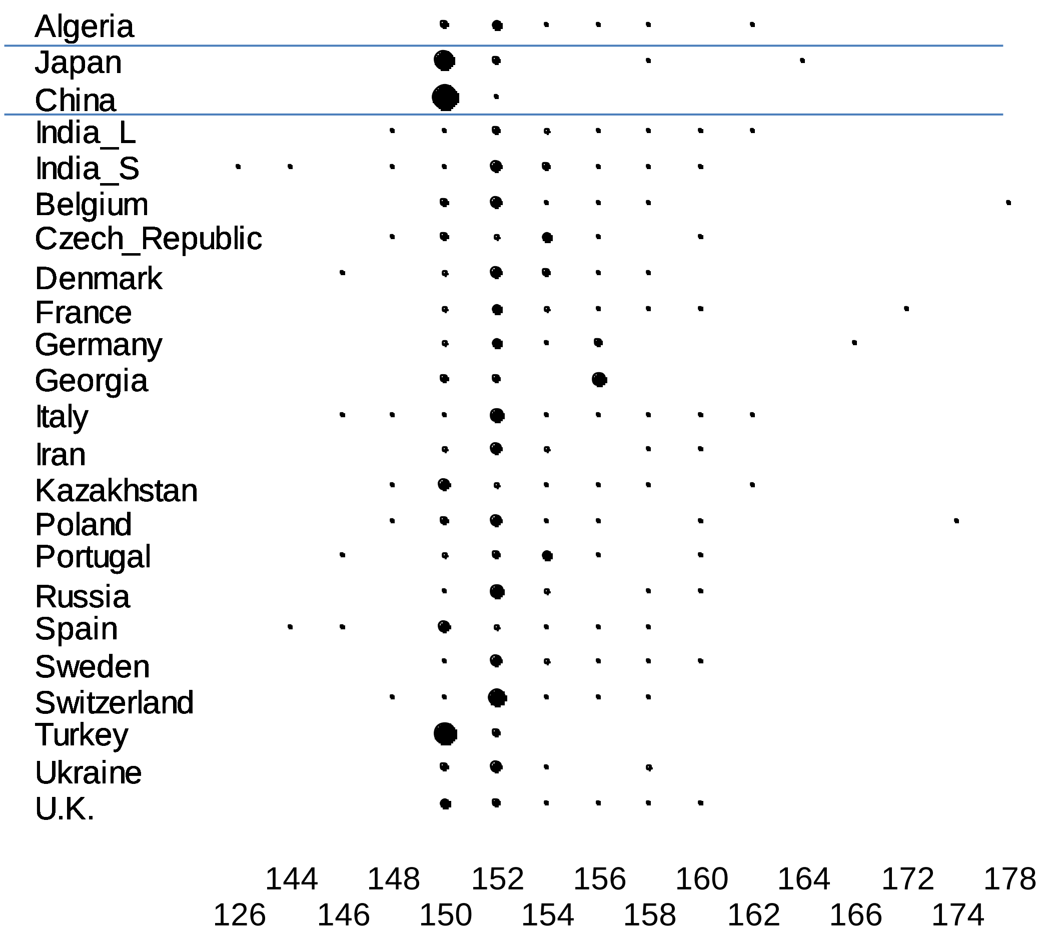

Locus di166 – 13 alleles

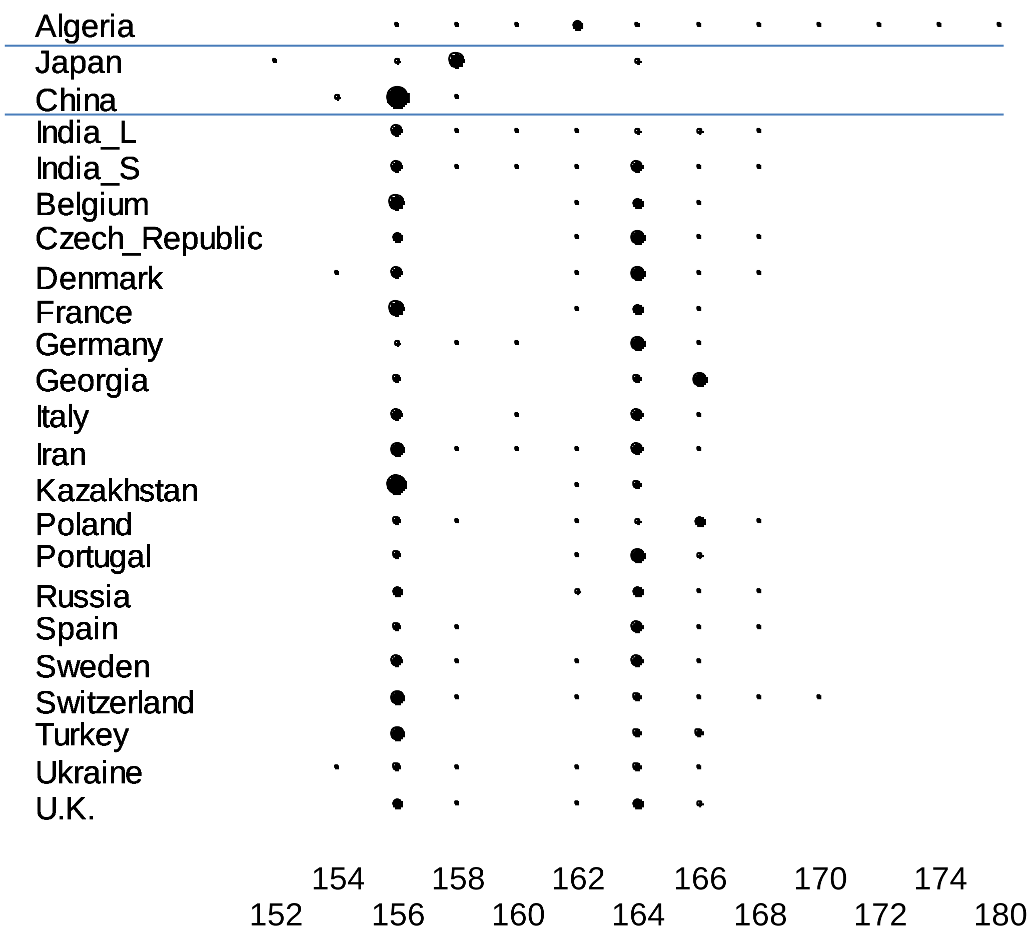

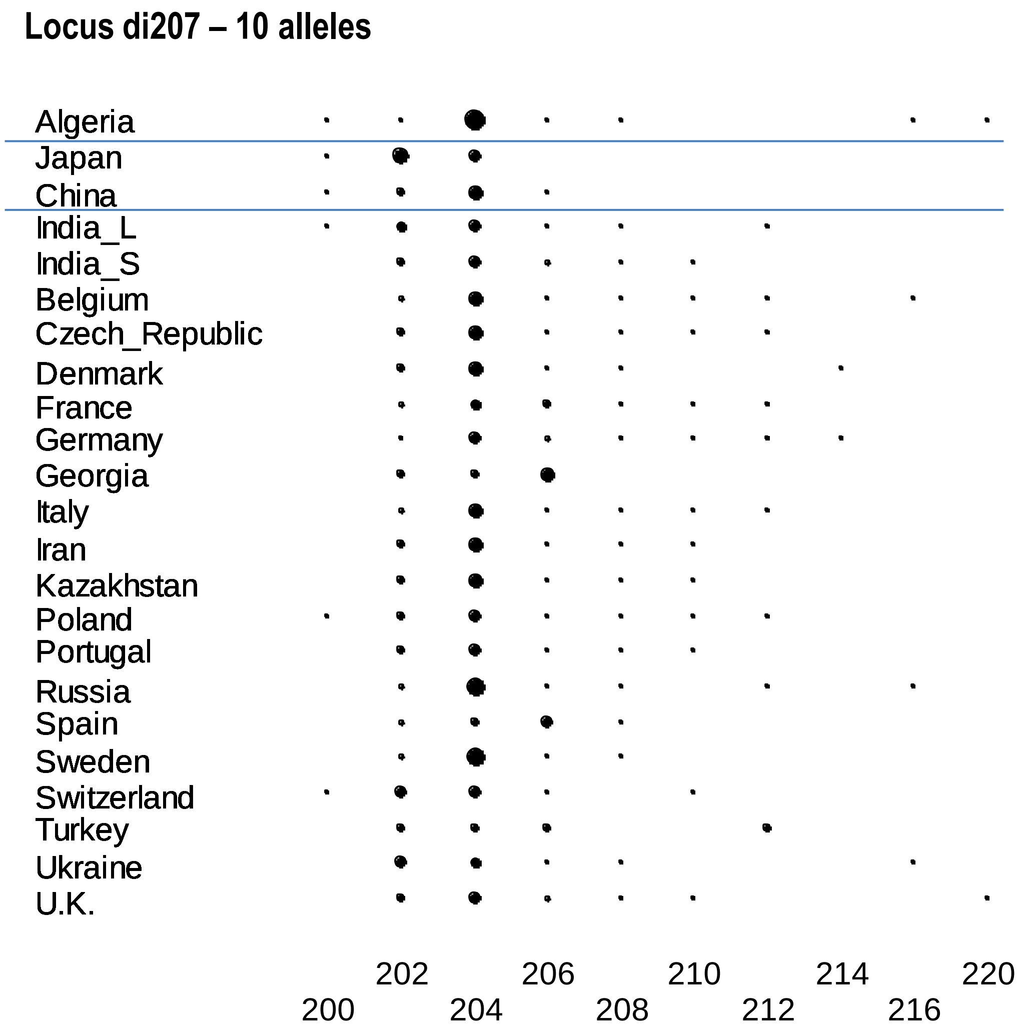
Locus di207 – 10 alleles
Algeria
Japan
China
India_L
India_S
Belgium
Czech_Republic
Denmark
France
Germany
Georgia
Italy
Iran
Kazakhstan
Poland
Portugal
Russia
Spain
Sweden
Switzerland
Turkey
Ukraine
U.K.
200
202
204
206
208
210
212
214
216
220

Locus di208 – 30 alleles

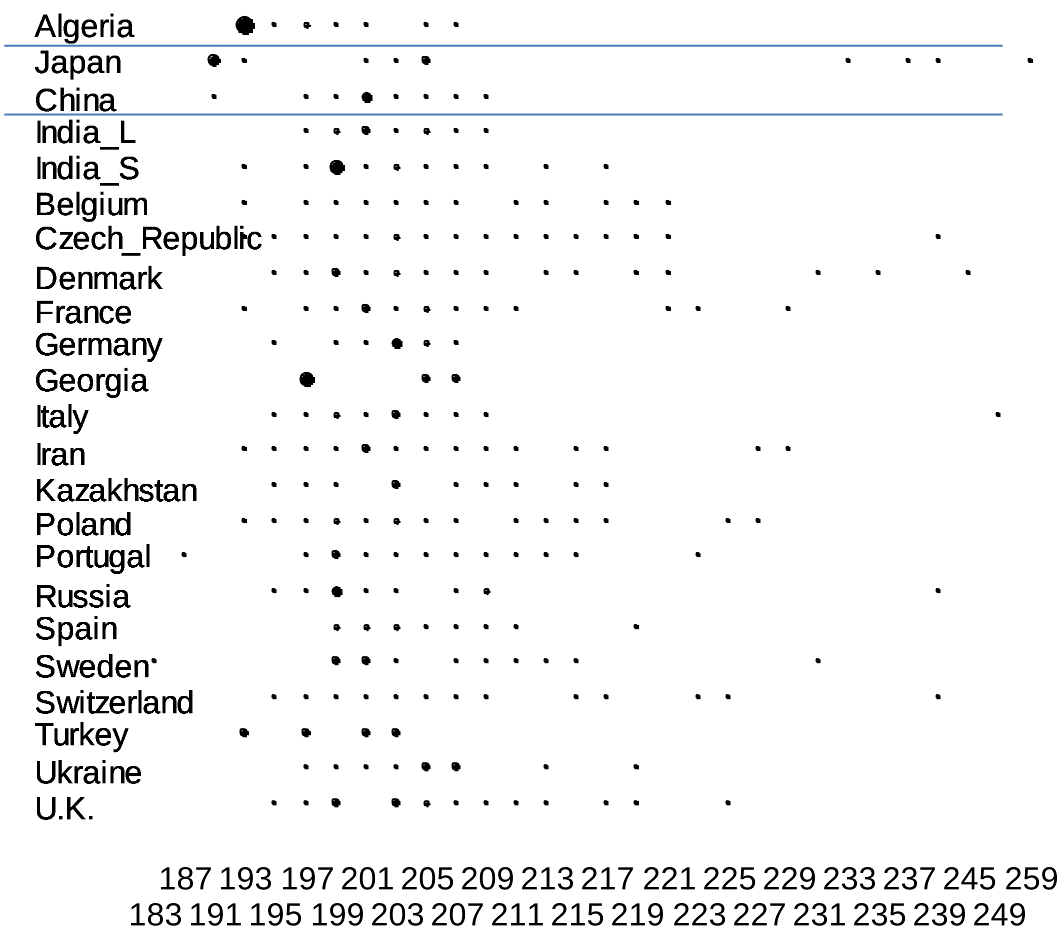

Locus di216 – 12 alleles

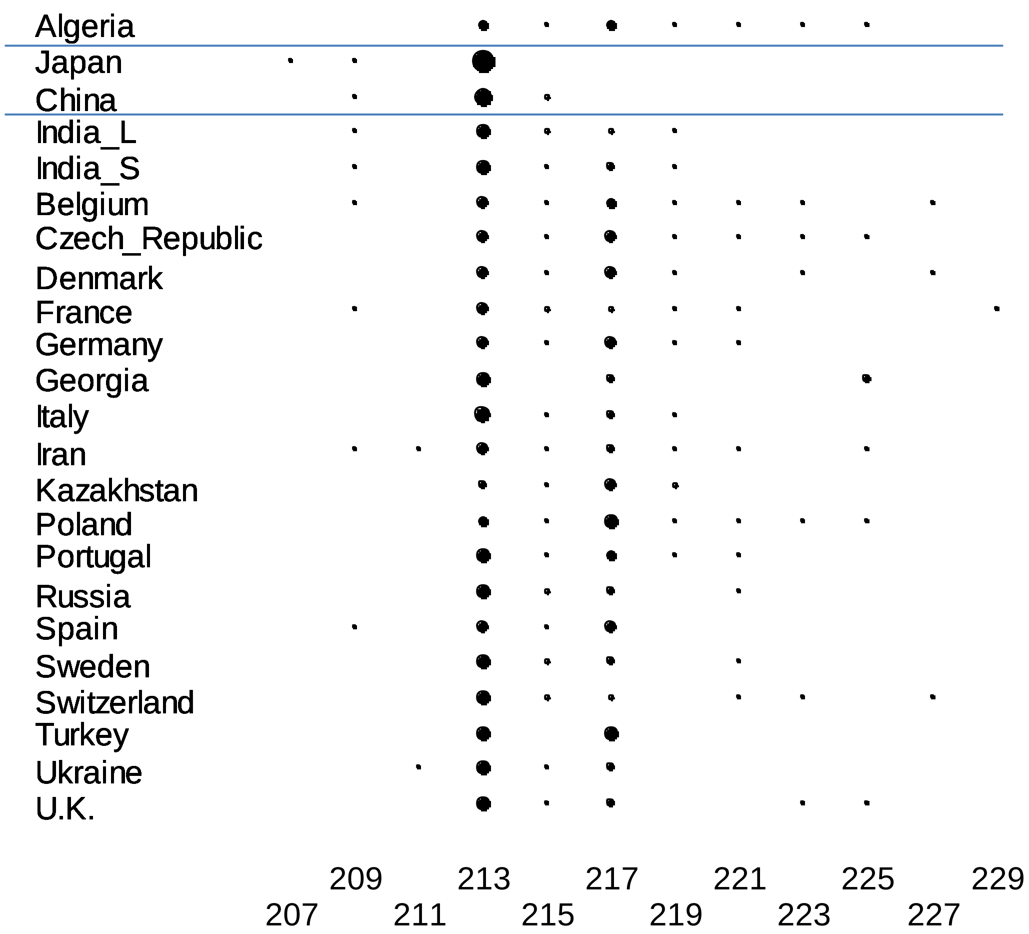

Locus di223 – 11 alleles

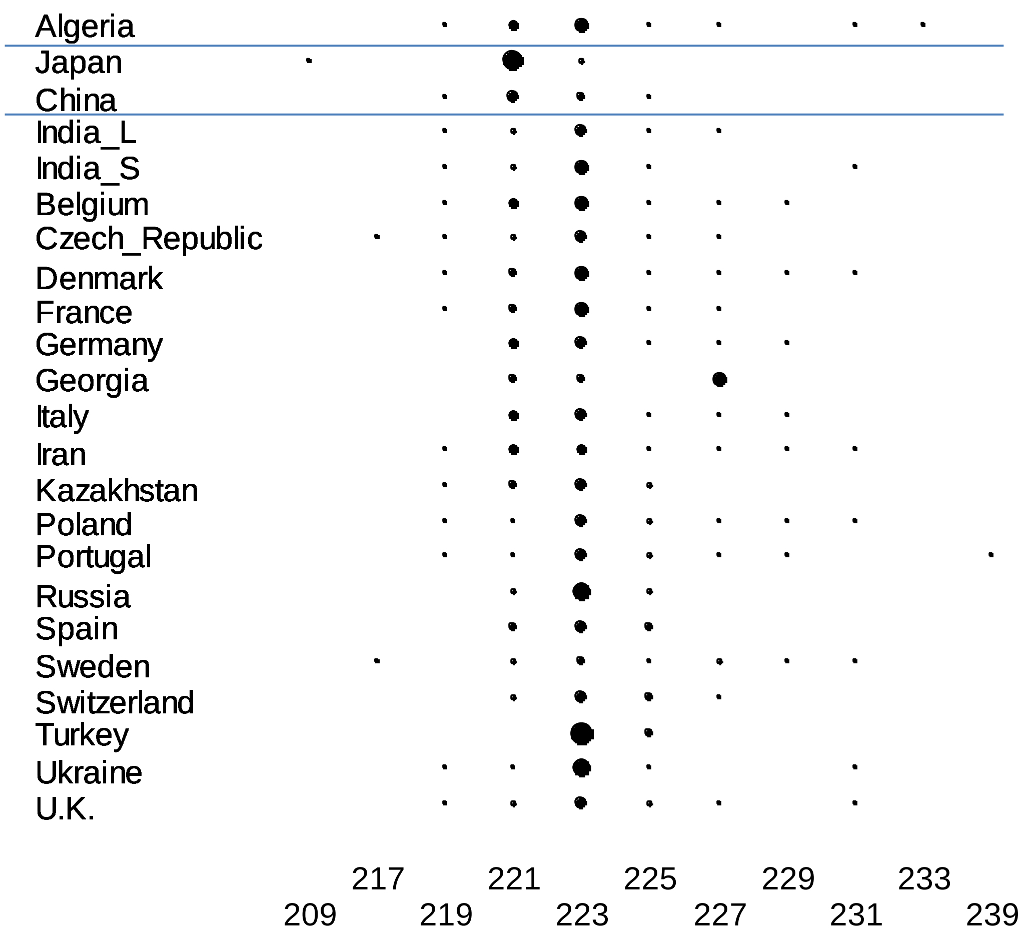

Locus di224 – 15 alleles

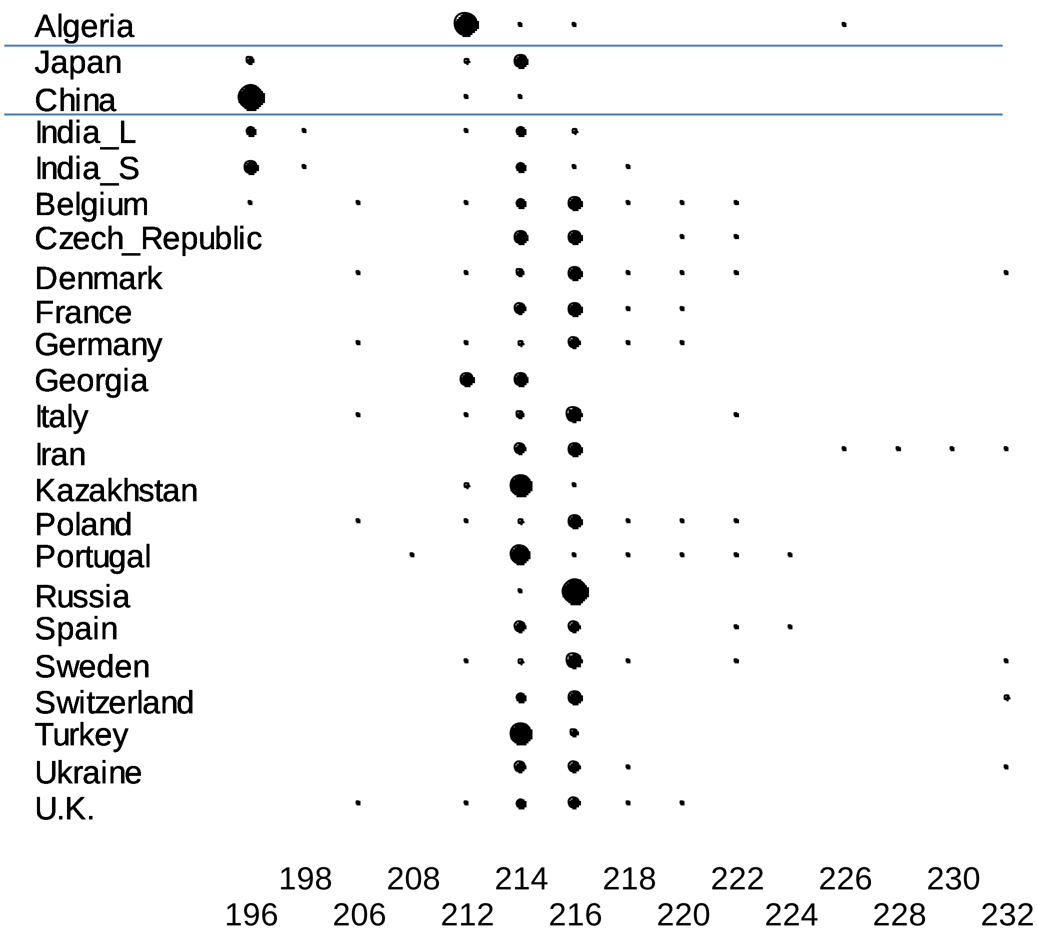

Locus di235 – 12 alleles

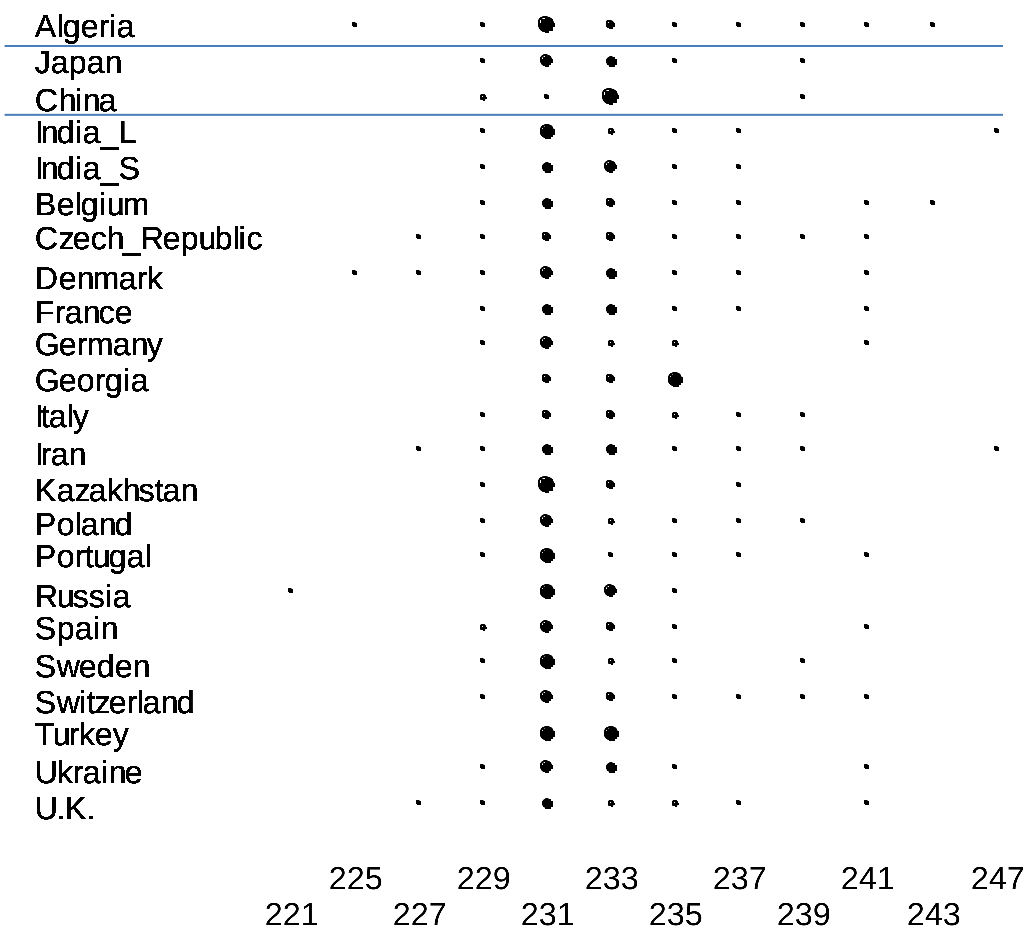

Locus di261 – 39 alleles

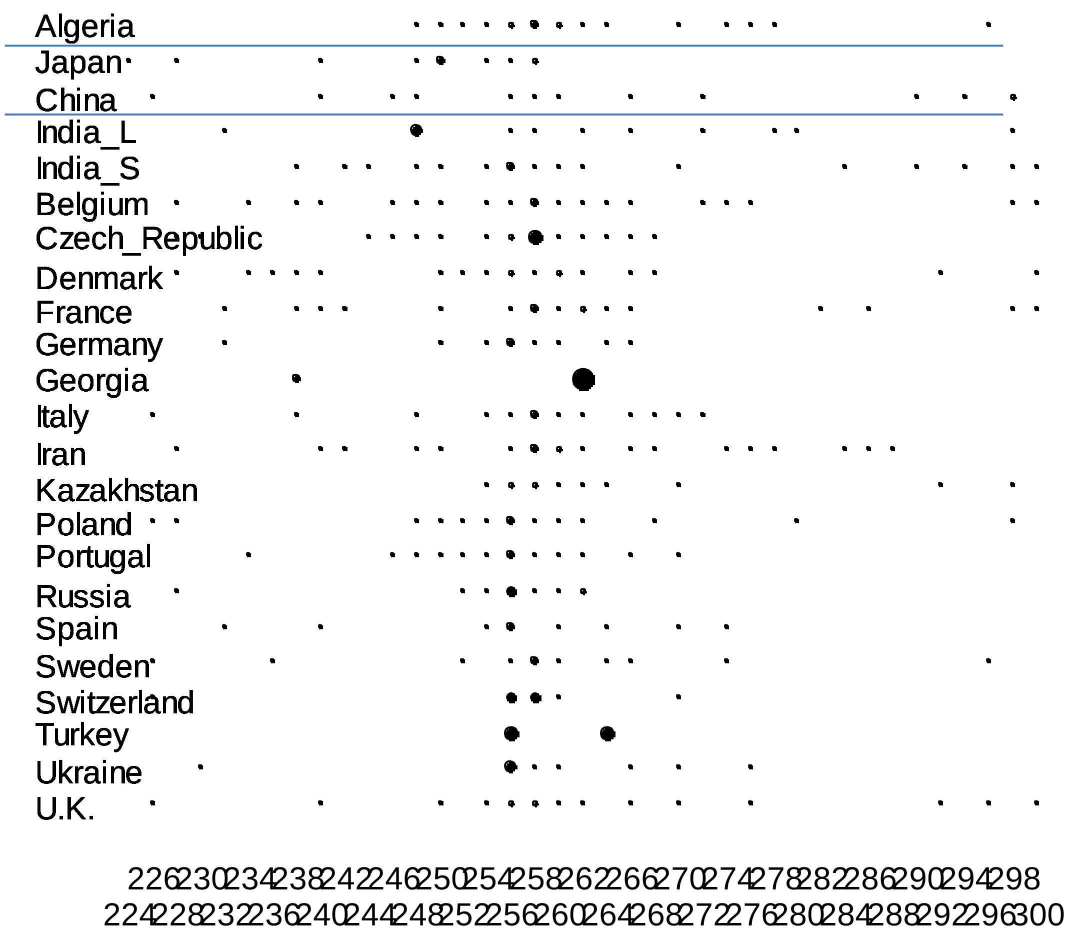

Locus di282 – 20 alleles

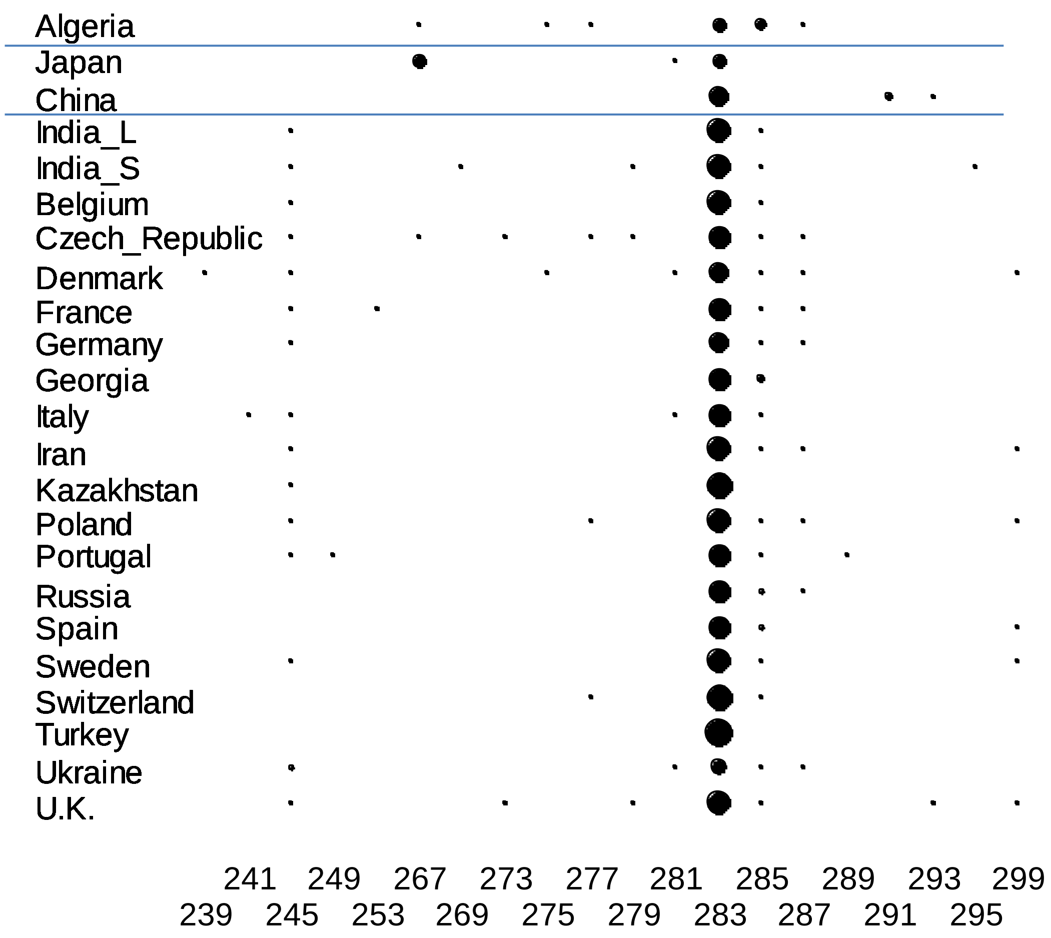

Locus di310 – 20 alleles

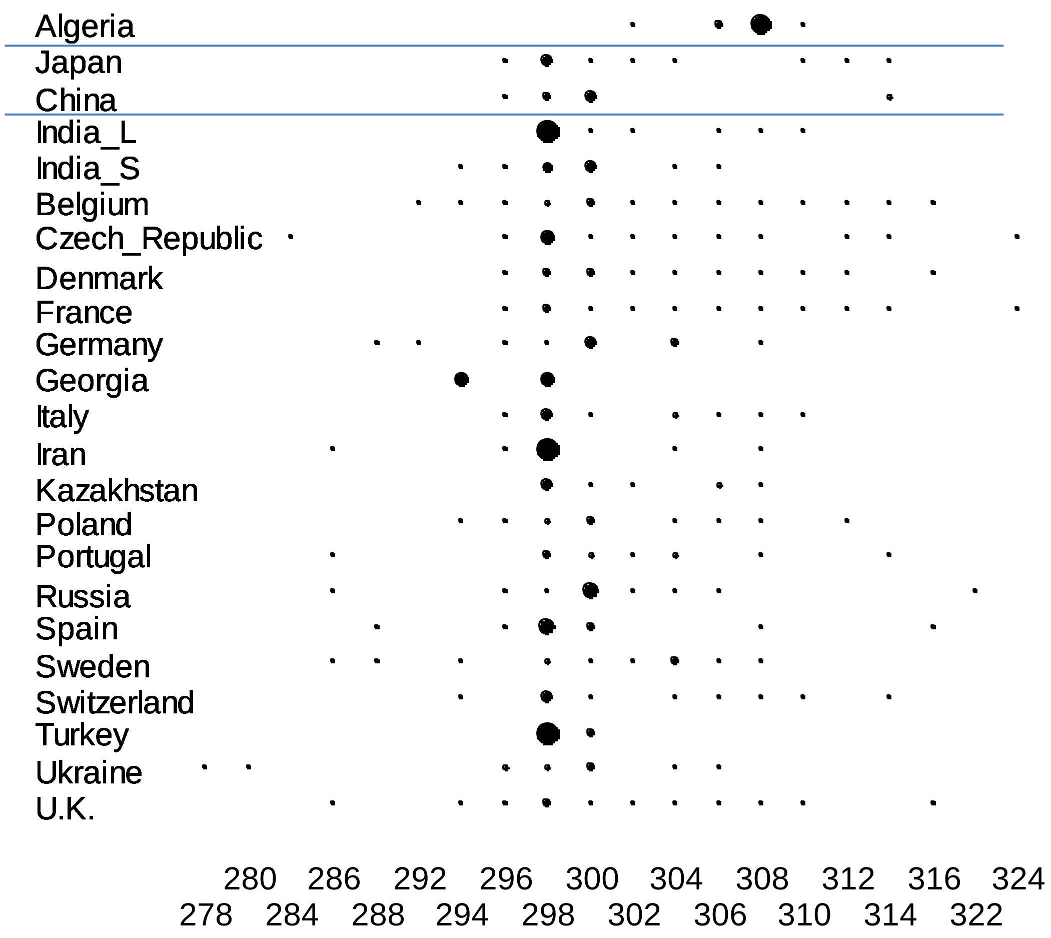

Locus di396 – 17 alleles

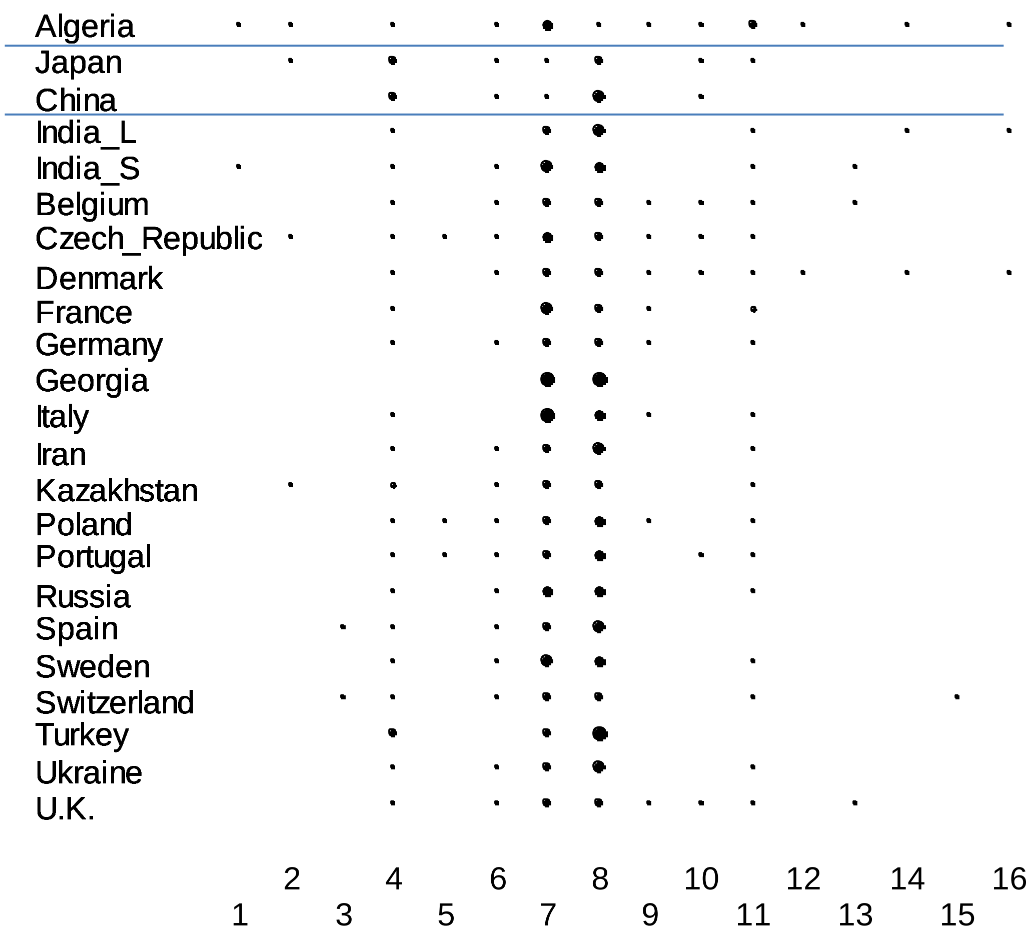

Locus tr158 – 8 alleles

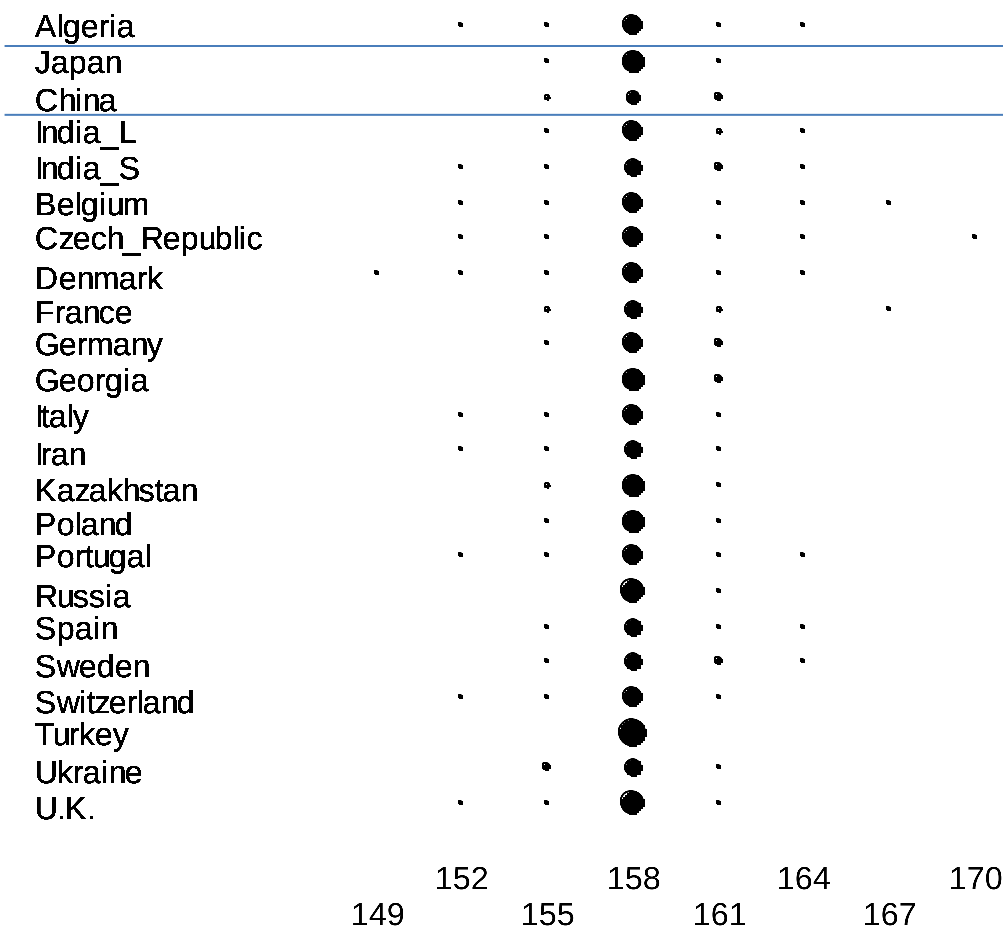

Locus te112 – 9 alleles

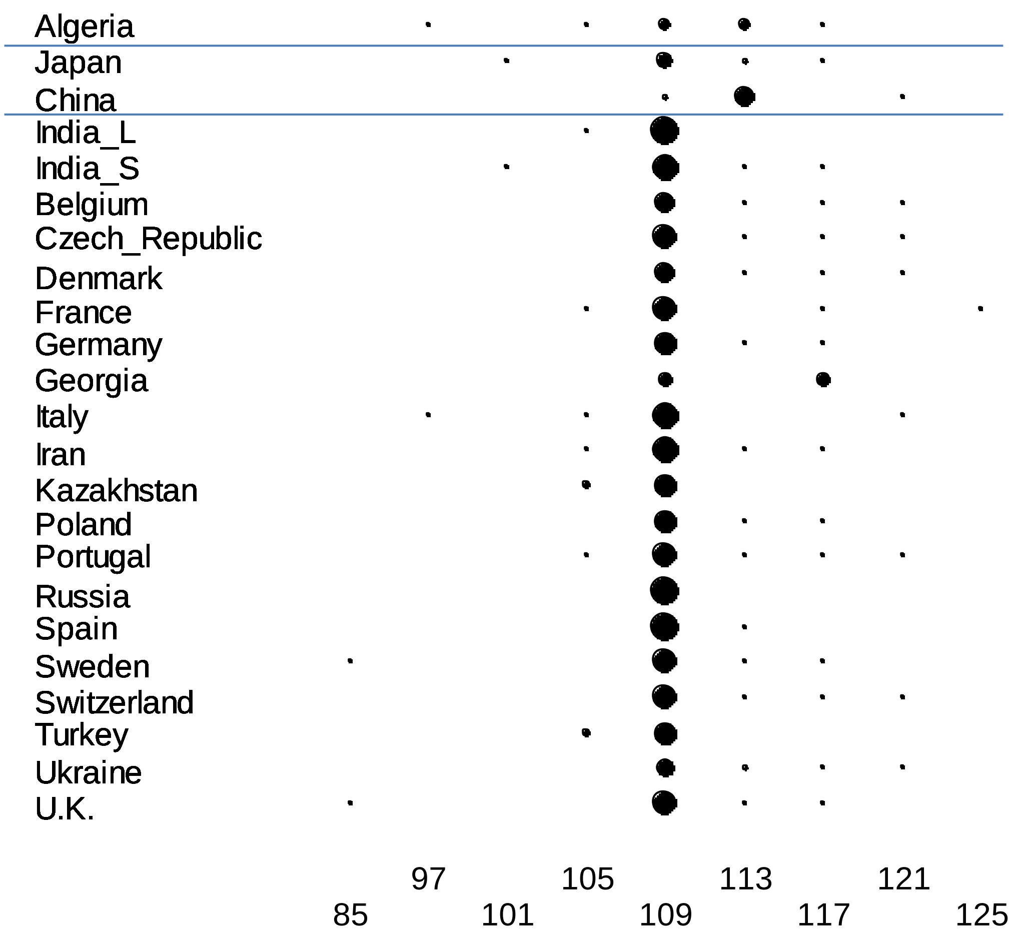

Locus te118 – 8 alleles

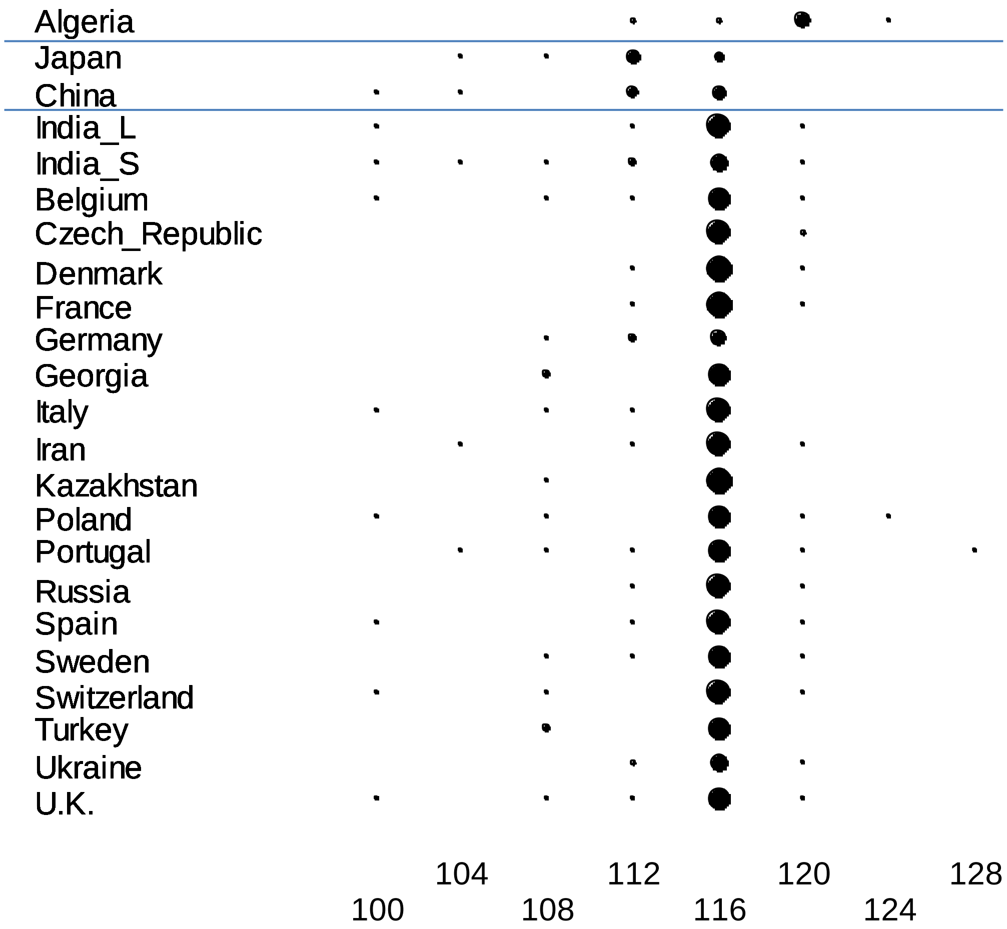

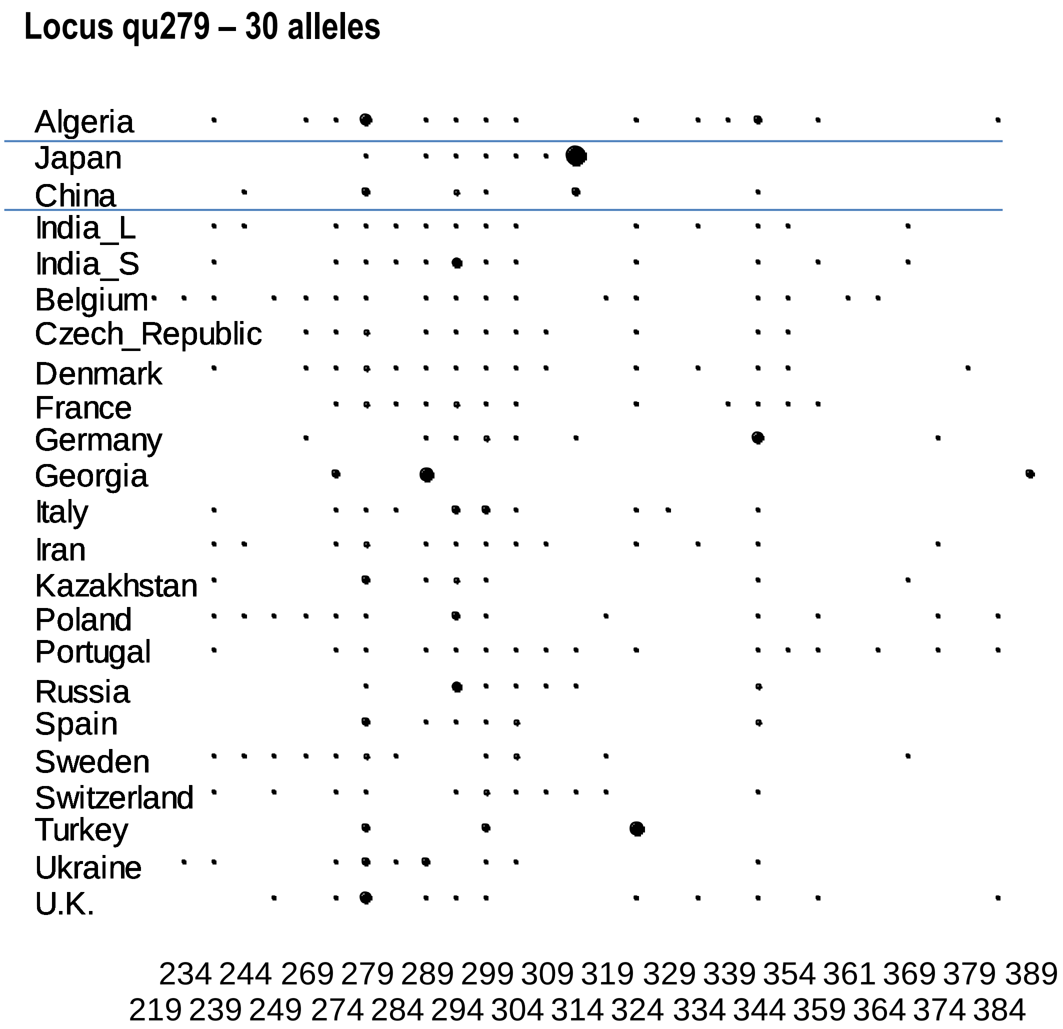
Locus qu279 – 30 alleles
Algeria
Japan
China
India_L
India_S
Belgium
Czech_Republic
Denmark
France
Germany
Georgia
Italy
Iran
Kazakhstan
Poland
Portugal
Russia
Spain
Sweden
Switzerland
Turkey
Ukraine
U.K.
234 244 269 279 289 299 309 319 329 339 354 361 369 379 389
219 239 249 274 284 294 304 314 324 334 344 359 364 374 384
