# Supplementary material for: Genetic diversity and structuring across the range of a widely distributed ladybird: focus on rear‐edge populations phenotypically divergent
Source: Ecol Evol. 2016 Jul 13;6(15):5517–29. doi: 10.1002/ece3.2288 (PMC4984522; doi:10.1002/ece3.2288)
Supplement: Supplementary file 2 — Figure S2. Results of the Bayesian structure analyses of Coccinella septempunctata populations across Palearctic, calculated with the program STRUCTURE. [file ECE3-6-5517-s002.pdf]

SUPPORTING INFORMATION

Genetic diversity and structuring across the range of a widely distributed ladybird: focus on rear-edge populations phenotypically divergent

Émilie LECOMPTE*, Mohand-Ameziane BOUANANI, Alexandra MAGRO & Brigitte CROUAU-ROY

Figure S2. Results of the Bayesian structure analyses of *C. septempunctata* populations across Paleartic, calculated with the program STRUCTURE. Estimation of the number of clusters (K) : (A) average values of ln P(K) resulting from a minimum of 15 runs for K = 1–15*,* with their corresponding standard deviation bars. (B) modal value of the second order rate of change of the likelihood function (ΔK), inferring that K = 3. (C) Bar plots, from K=2 to K=10, showing the proportion of individual multilocus genotypes assigned to each of the clusters, illustrated by the different colors. The vertical lines separate the 28 sampled populations, included in this graph *a posteriori*.

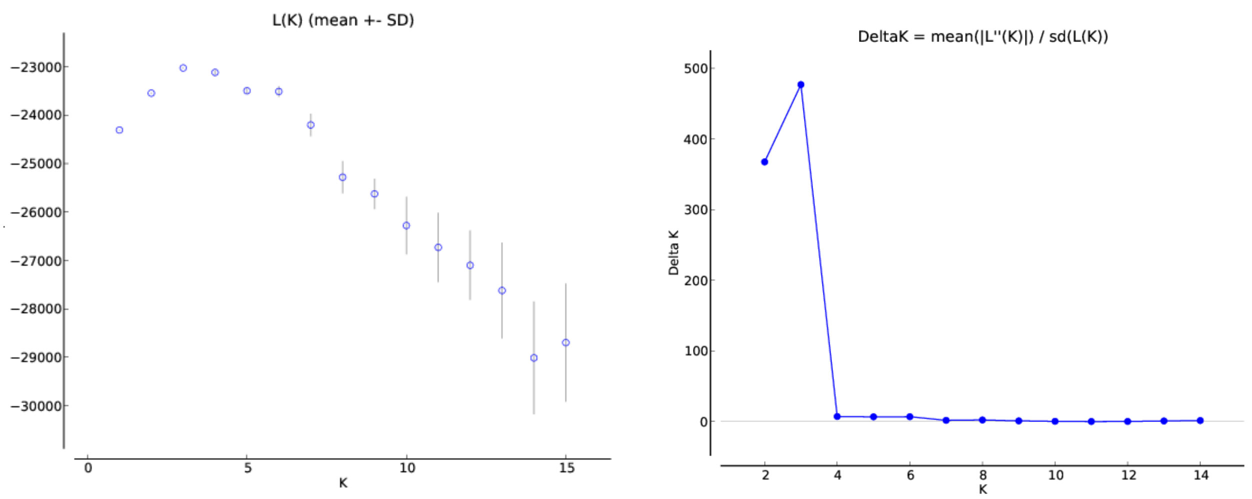

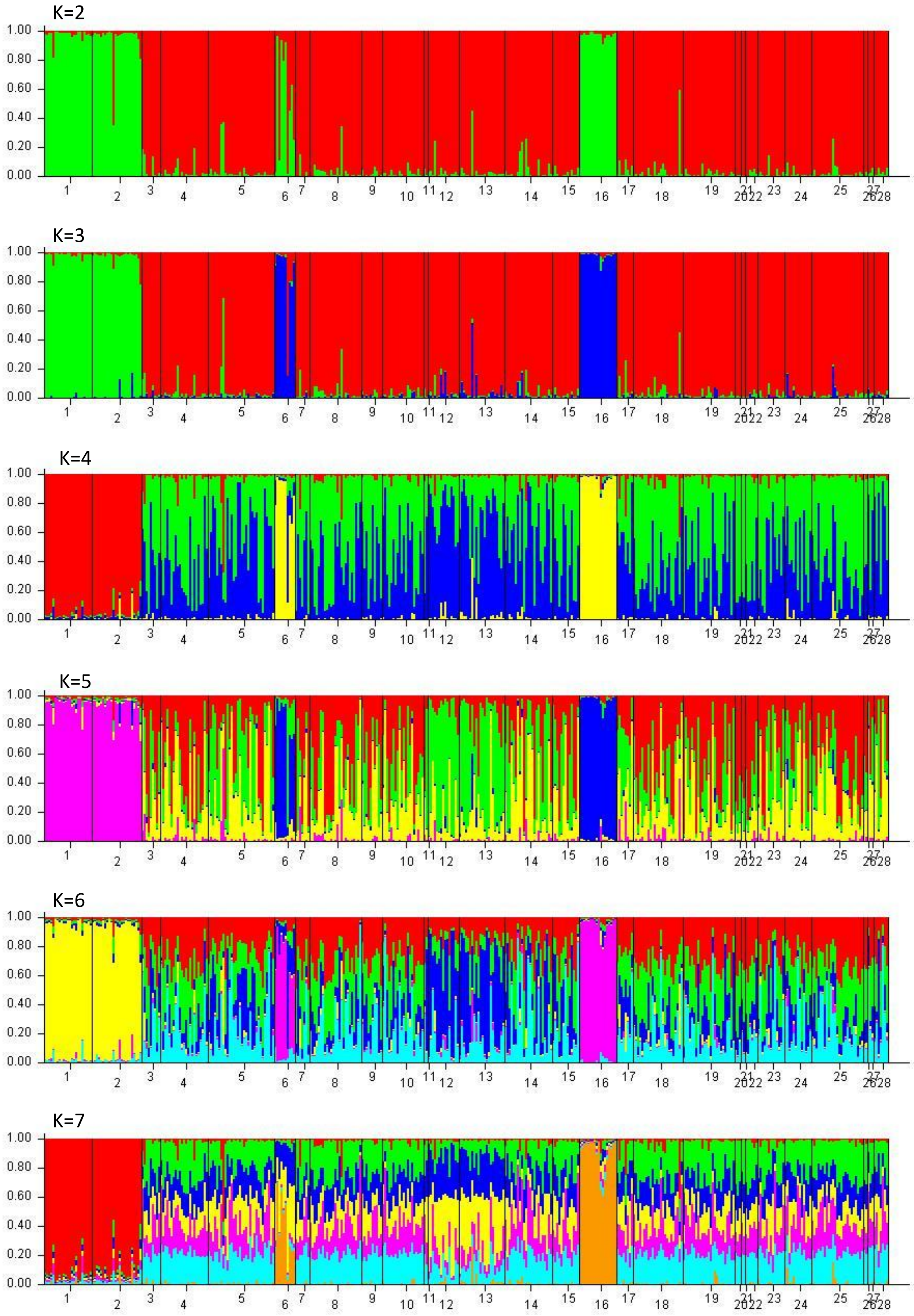
K=2
K=3
K=4
K=5
K=6
K=7
1.00
0.80
0.60
0.40
0.20
0.00
1 2 3 4 5 6 7 8 9 10 11 12 13 14 15 16 17 18 19 20 21 22 23 24 25 26 27 28

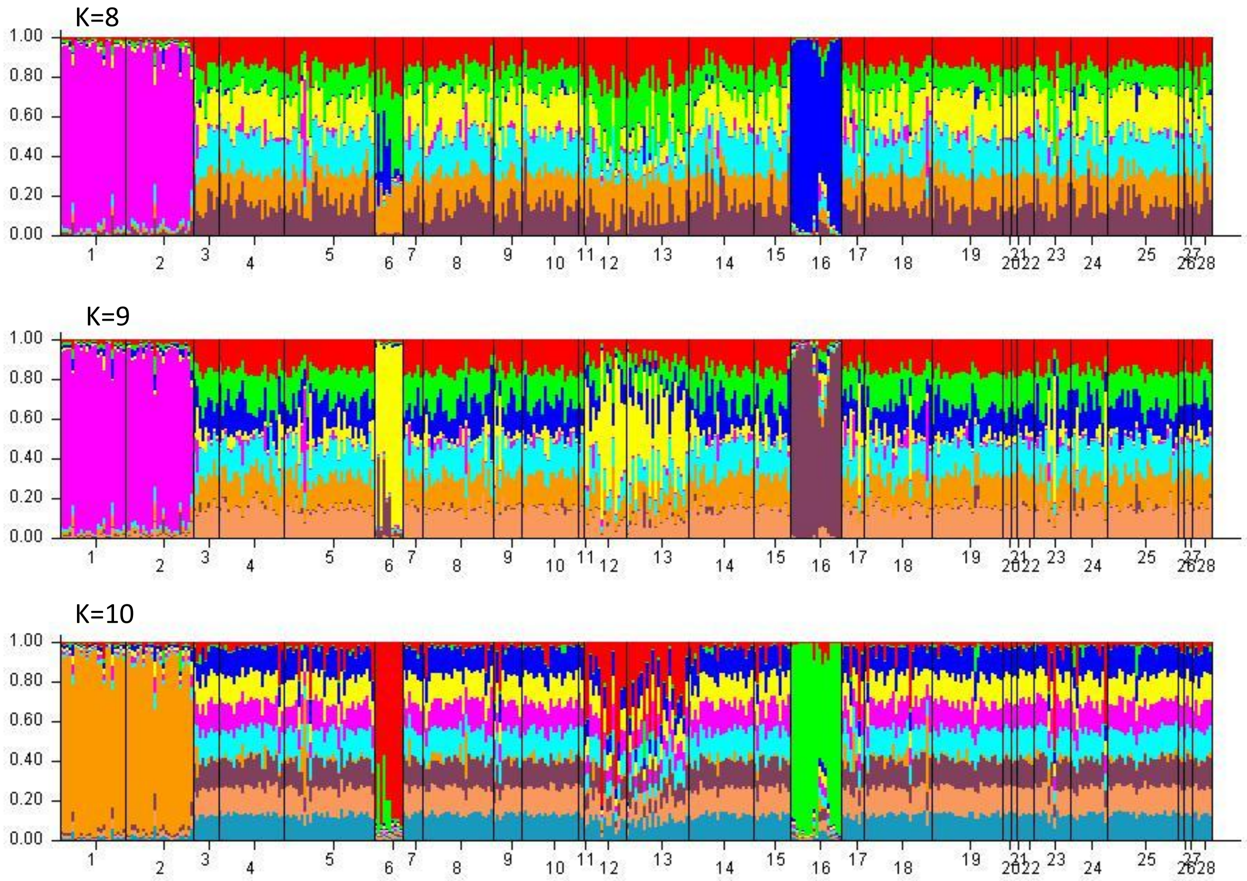

Populations: **1 Algeria, Alger; 2 Algeria, Biskra**; 3 Germany, Groß Lüsewitz; 4 United Kingdom, Norwich; 5 Belgium, Gembloux; **6 China**, Chengdu; 7 Denmark, Aarhus; 8 Denmark, Skagen; 9 Spain, Victoria-Gasteiz; 10 France, Toulouse; 11 Georgia, Doesi; 12 India, Lucknow City; 13 India, Shimla; 14 Iran, Saveh; 15 Italy, Perugia; **16 Japan**, Tsuruoka; 17 Kazakhstan, Kasskelen; 18 Poland, Tomianski; 19 Portugal, Lisbon; 20 Russia, Aydar 21 Russia, Borisovka 22 Russia, Orekovo Zuevo 23 Sweden, Alnarp; 24 Switzerland, Delemont; 25 Czech Republic, Prague; 26 Turkey, Izmir; 27 Ukraine, Odessa; 28 Ukraine, Prymors'kyi
