# Supplementary material for: Genetic diversity and structuring across the range of a widely distributed ladybird: focus on rear‐edge populations phenotypically divergent
Source: Ecol Evol. 2016 Jul 13;6(15):5517–29. doi: 10.1002/ece3.2288 (PMC4984522; doi:10.1002/ece3.2288)

SUPPORTING INFORMATION

Genetic diversity and structuring across the range of a widely distributed ladybird: focus on rear-edge populations phenotypically divergent

Émilie LECOMPTE*, Mohand-Ameziane BOUANANI, Alexandra MAGRO & Brigitte CROUAU-ROY

Figure S3. DAPC assignment: posterior probability of assignment of each individual to the five DAPC clusters.

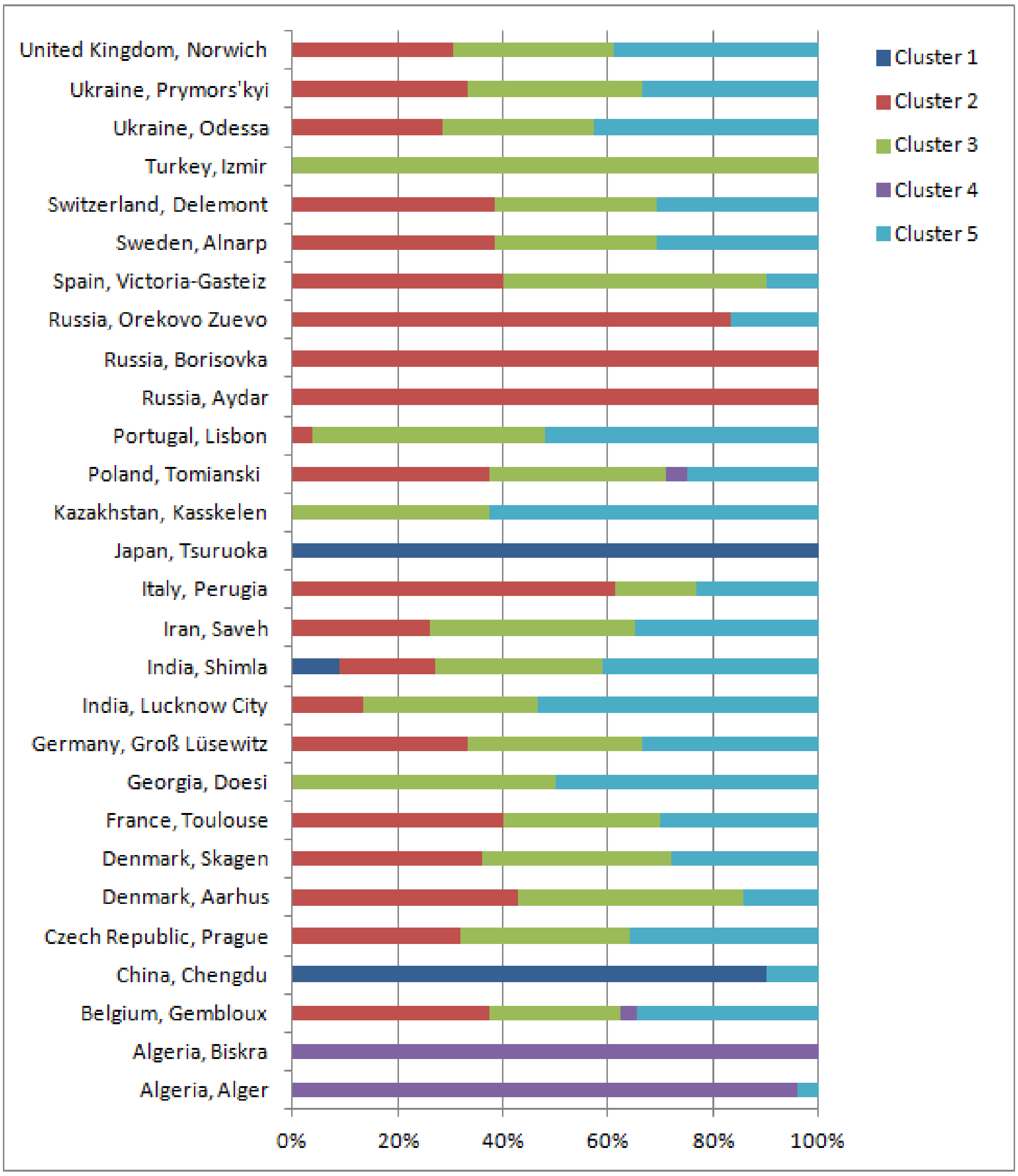

Supplement: Supplementary file 3 — Figure S3. DAPC assignment: posterior probability of assignment of each individual to the five DAPC clusters. [file ECE3-6-5517-s003.pdf]
