# Supplementary material for: Genetic diversity and structuring across the range of a widely distributed ladybird: focus on rear‐edge populations phenotypically divergent
Source: Ecol Evol. 2016 Jul 13;6(15):5517–29. doi: 10.1002/ece3.2288 (PMC4984522; doi:10.1002/ece3.2288)
Supplement: Supplementary file 4 — Table S1. F IS values, per population and per locus, and across all loci with the significant values indicated in bold. [file ECE3-6-5517-s004.docx]

Genetic diversity and structuring across the range of a widely distributed ladybird:
focus on rear-edge populations phenotypically divergent

**Table S1.** F_IS_ values, per population and per locus, and across all loci with the significant values indicated in bold.

| **Population \ Locus** | **di130** | **di154** | **di155** | **di166** | **di207** | **di208** | **di216** | **di223** | **di224** | **di235** | **di261** | **di282** | **di310** | **di396** | **tr158** | **te112** | **te118** | **qu279** | **All loci** |
| --- | --- | --- | --- | --- | --- | --- | --- | --- | --- | --- | --- | --- | --- | --- | --- | --- | --- | --- | --- |
| **Algeria, Alger** | 0.269 | 0.227 | 0.567 | 0.411 | 0.271 | -0.130 | 0.502 | 0.137 | -0.158 | 0.212 | 0.255 | -0.615 | 0.529 | 0.339 | -0.090 | 0.181 | 0.569 | 0.146 | **0.219** |
| **Algeria, Biskra** | 0.092 | 0.446 | 0.282 | 0.268 | -0.021 | -0.053 | 0.157 | 0.005 | 0.007 | -0.108 | 0.461 | -0.596 | 0.926 | 0.118 | 0.330 | 0.359 | 0.156 | 0.028 | **0.169** |
| **Belgium, Gembloux** | 0.406 | 0.635 | 0.573 | 0.076 | 0.201 | 0.167 | -0.291 | 0.515 | 0.535 | 0.038 | 0.435 | -0.028 | 0.777 | 0.164 | 0.051 | 0.045 | -0.137 | 0.423 | **0.301** |
| **China, Chengdu** | -0.096 | 0.438 | 1.000 | 0.289 | -0.050 | -0.059 | -0.313 | 0.450 | -0.029 | -0.231 | 0.041 | -0.301 | 0.873 | 0.191 | 0.085 | 0.273 | 0.027 | 0.154 | 0.139 |
| **Czech Republic, Prague** | 0.326 | 0.606 | -0.057 | 0.120 | 0.072 | 0.068 | -0.287 | 0.420 | 0.112 | 0.001 | 0.494 | -0.160 | 0.572 | 0.005 | 0.037 | -0.148 | 0.341 | 0.193 | **0.168** |
| **Denmark, Aarhus** | 0.429 | 0.662 | -0.029 | -0.333 | 0.127 | 0.304 | -0.333 | 0.520 | 0.250 | 0.077 | 0.850 | -0.171 | 1.000 | 0.143 | -0.231 | 0.379 | 0.000 | 0.514 | **0.289** |
| **Denmark, Skagen** | 0.092 | 0.608 | 0.252 | 0.034 | -0.095 | 0.094 | -0.210 | 0.309 | 0.385 | 0.098 | 0.423 | -0.202 | 0.538 | 0.397 | -0.120 | -0.168 | -0.029 | 0.308 | **0.200** |
| **France, Toulouse** | 0.294 | 0.345 | 0.501 | -0.118 | 0.012 | 0.024 | -0.264 | 0.633 | 0.381 | 0.075 | 0.387 | -0.191 | 0.509 | 0.186 | -0.029 | -0.165 | -0.059 | 0.333 | **0.199** |
| **Germany, Groß Lüsewitz** | 0.135 | 0.724 | 0.453 | 0.059 | 0.034 | 0.344 | -0.255 | 0.423 | 0.127 | -0.219 | 0.298 | -0.026 | 0.571 | 0.130 | -0.111 | -0.143 | 0.111 | 0.231 | **0.189** |
| **India, Lucknow city** | 0.168 | 0.500 | 0.694 | 0.114 | -0.007 | 0.524 | -0.338 | 0.205 | 0.424 | 0.268 | 0.270 | -0.129 | 0.138 | -0.151 | 0.030 | 0.000 | 0.067 | 0.296 | **0.224** |
| **India, Shimla** | 0.163 | 0.576 | 0.388 | 0.171 | -0.018 | 0.159 | -0.481 | 0.688 | 0.596 | 0.017 | 0.369 | -0.105 | 0.437 | 0.074 | -0.209 | 0.323 | 0.007 | 0.429 | **0.224** |
| **Iran, Saveh** | 0.349 | 0.599 | -0.139 | -0.251 | 0.061 | 0.231 | -0.306 | 0.271 | 0.261 | 0.111 | 0.238 | -0.135 | 0.618 | 0.123 | -0.051 | -0.078 | 0.060 | 0.523 | **0.168** |
| **Italy, Perugia** | 0.573 | 0.628 | 0.092 | -0.137 | -0.263 | 0.108 | -0.091 | 0.575 | 0.505 | 0.040 | 0.579 | -0.191 | 0.622 | -0.348 | 0.241 | -0.043 | -0.121 | 0.289 | **0.215** |
| **Japan, Tsuruoka** | -0.070 | 0.109 | 0.049 | 0.380 | -0.094 | 0.214 | -0.193 | 0.016 | -0.199 | 0.431 | 0.137 | -0.388 | 0.172 | -0.094 | -0.193 | 0.009 | -0.071 | -0.037 | 0.033 |
| **Kazakhstan, Kasskelen** | 0.195 | 0.189 | 0.385 | 0.250 | -0.105 | 0.192 | -0.195 | 0.494 | -0.191 | 0.200 | 0.611 | 0.000 | 0.829 | 0.622 | 0.429 | 0.391 | 0.000 | 0.725 | **0.331** |
| **Poland, Tomianski** | 0.003 | 0.075 | 0.100 | -0.211 | -0.234 | -0.012 | -0.384 | 0.274 | 0.251 | -0.282 | 0.668 | -0.110 | 0.406 | 0.244 | -0.107 | 0.292 | -0.020 | 0.234 | 0.085 |
| **Portugal, Lisbon** | 0.050 | 0.288 | 0.211 | 0.179 | 0.259 | 0.155 | -0.321 | 0.197 | 0.272 | -0.003 | 0.486 | -0.198 | 0.465 | 0.110 | -0.025 | -0.100 | 0.065 | 0.263 | **0.165** |
| **Spain, Victoria-Gasteiz** | 0.036 | 0.791 | 0.388 | 0.217 | -0.116 | 0.100 | -0.309 | 0.571 | 0.707 | 0.357 | 0.669 | 0.289 | 0.405 | -0.009 | 0.481 | 0.000 | 0.463 | 0.772 | **0.362** |
| **Sweden, Alnarp** | 0.085 | 0.547 | 0.197 | 0.103 | -0.187 | 0.063 | -0.378 | 0.639 | 0.431 | 0.158 | 0.532 | -0.121 | 0.101 | -0.067 | 0.326 | -0.121 | -0.021 | 0.229 | **0.179** |
| **Switzerland, Delemont** | 0.579 | 0.557 | 0.503 | -0.173 | 0.018 | 0.102 | -0.106 | 0.481 | 0.423 | -0.017 | 0.783 | -0.059 | 0.894 | 0.219 | -0.231 | -0.079 | -0.079 | 0.502 | **0.297** |
| **Ukraine, Prymors'kyi** | 0.806 | 0.520 | 0.419 | 0.829 | 0.000 | 0.040 | -0.364 | 0.769 | 0.793 | -0.355 | 0.304 | -0.154 | 0.532 | -0.200 | -0.231 | -0.111 | 0.122 | 0.392 | **0.267** |
| **United Kingdom, Norwich** | 0.456 | 0.399 | 0.434 | 0.017 | 0.043 | 0.321 | -0.284 | 0.426 | 0.230 | 0.146 | 0.473 | -0.097 | 0.658 | 0.491 | -0.011 | -0.125 | 0.053 | 0.528 | **0.284** |
