# Supplementary material for: Genetic diversity and structuring across the range of a widely distributed ladybird: focus on rear‐edge populations phenotypically divergent
Source: Ecol Evol. 2016 Jul 13;6(15):5517–29. doi: 10.1002/ece3.2288 (PMC4984522; doi:10.1002/ece3.2288)
Supplement: Supplementary file 5 — Table S2. Null allele frequencies estimated per locus and per population. “–” means that null alleles were not encountered. [file ECE3-6-5517-s005.docx]

Genetic diversity and structuring across the range of a widely distributed ladybird:
focus on rear-edge populations phenotypically divergent

**Table S2**. Null allele frequencies estimated per locus and per population, using FreeNA. “-“ means that null alleles were not encountered.

| **Population \ locus** | **di130** | **di154** | **di155** | **di166** | **di207** | **di208** | **di216** | **di223** | **di224** | **di235** | **di261** | **di282** | **di310** | **di396** | **tr158** | **te112** | **te118** | **qu279** | **Mean across loci** |
| --- | --- | --- | --- | --- | --- | --- | --- | --- | --- | --- | --- | --- | --- | --- | --- | --- | --- | --- | --- |
| **Algeria, Alger** | 8,0% | 10,0% | 24,6% | 16,3% | 9,7% | - | 20,4% | - | - | 2,8% | 10,6% | - | 14,5% | 11,5% | - | 3,3% | 18,3% | - | **8%** |
| **Algeria, Biskra** | 2,4% | 18,3% | 10,8% | 12,1% | - | - | 6,3% | 2,2% | - | - | 19,3% | - | 32,7% | - | 11,7% | 14,2% | 6,2% | - | **8%** |
| **Belgium, Gembloux** | 16,3% | 28,9% | 23,5% | - | 8,1% | 6,3% | - | 19,5% | 20,0% | 2,2% | 18,8% | - | 35,1% | 2,7% | - | - | - | 19,2% | **11%** |
| **China, Chengdu** | - | 18,0% | 20,4% | 8,0% | - | - | - | 17,4% | - | - | - | - | 35,9% | 7,2% | - | 7,3% | - | 3,4% | **7%** |
| **Czech Republic, Prague** | 12,5% | 25,5% | - | 0,8% | 4,9% | - | - | 16,7% | 1,3% | - | 21,1% | - | 24,7% | 2,0% | 3,9% | - | 9,4% | 8,0% | **7%** |
| **Denmark, Aarhus** | 16,6% | 27,0% | - | - | - | 8,6% | - | 14,4% | 10,7% | - | 37,5% | - | 41,1% | 2,2% | - | 13,5% | - | 20,7% | **11%** |
| **Denmark, Skagen** | 4,7% | 26,7% | 9,2% | - | - | 1,6% | - | 12,8% | 14,7% | - | 18,3% | - | 24,0% | 14,1% | - | - | - | 13,9% | **8%** |
| **France, Toulouse** | 11,7% | 15,0% | 20,5% | - | - | - | - | 24,9% | 11,6% | 1,0% | 16,5% | - | 23,2% | 4,3% | - | - | - | 13,6% | **8%** |
| **Germany, Groß Lüsewitz** | 2,6% | 29,3% | 18,2% | 1,8% | - | 11,6% | - | 16,1% | 2,6% | - | 8,8% | - | 19,4% | - | - | - | 4,7% | 4,2% | **7%** |
| **India, Lucknow city** | 1,5% | 20,8% | 30,6% | - | - | 21,7% | - | 8,8% | 16,2% | 8,7% | 8,7% | - | 7,3% | - | - | - | - | 12,2% | **8%** |
| **India, Shimla** | 5,1% | 25,4% | 15,0% | 2,2% | - | 5,4% | - | 28,4% | 23,1% | - | 16,4% | - | 15,9% | 6,5% | - | - | - | 19,1% | **9%** |
| **Iran, Saveh** | 14,2% | 27,2% | - | - | 1,6% | 9,7% | - | 11,1% | 11,8% | 2,1% | 9,5% | - | 20,6% | 2,7% | - | - | - | 23,5% | **7%** |
| **Italy, Perugia** | 21,1% | 26,4% | - | - | - | 3,1% | - | 22,9% | 16,7% | - | 26,6% | - | 25,9% | - | 6,0% | - | - | 9,5% | **9%** |
| **Japan, Tsuruoka** | - | 5,6% | - | 14,8% | - | 2,1% | - | - | - | 15,2% | 9,2% | - | 7,7% | 1,1% | - | - | 1,7% | - | **3%** |
| **Kazakhstan, Kasskelen** | 1,5% | - | 10,3% | 6,3% | - | 5,4% | - | 18,2% | - | 1,9% | 26,5% | - | 33,8% | 23,0% | 12,4% | 10,4% | - | 31,3% | **10%** |
| **Poland, Tomianski** | 1,6% | 3,3% | 2,3% | - | - | - | - | 12,1% | 8,1% | - | 30,2% | - | 18,3% | 10,1% | - | 8,8% | - | 9,3% | **6%** |
| **Portugal, Lisbon** | 3,4% | 11,6% | 10,4% | 5,4% | 9,8% | 5,6% | - | 4,4% | 10,5% | 1,4% | 22,5% | - | 19,2% | 1,6% | - | - | - | 11,3% | **7%** |
| **Spain, Victoria-Gasteiz** | 2,6% | 36,0% | 13,1% | 9,8% | - | - | - | 21,7% | 26,4% | 14,0% | 29,4% | 8,0% | 12,4% | 5,4% | 18,0% | - | 14,4% | 33,9% | **14%** |
| **Sweden, Alnarp** | - | 23,6% | 7,9% | - | - | 1,8% | - | 27,3% | 15,7% | 8,7% | 22,6% | - | - | - | 8,8% | - | - | 10,0% | **7%** |
| **Switzerland, Delemont** | 23,1% | 24,5% | 20,0% | - | - | 4,1% | - | 18,0% | 14,5% | - | 31,8% | - | 39,2% | 4,4% | - | - | - | 21,8% | **11%** |
| **United Kingdom, Norwich** | 19,1% | 18,3% | 16,9% | - | 0,5% | 12,8% | - | 17,3% | 7,9% | 5,8% | 22,6% | - | 29,9% | 20,0% | - | - | 3,5% | 22,9% | **11%** |
| **Mean across populations** | **7,4%** | **20,2%** | **11,8%** | **3,9%** | **1,7%** | **4,3%** | **1,3%** | **14,8%** | **10,2%** | **2,9%** | **19,2%** | **0,4%** | **22,5%** | **5,0%** | **3,0%** | **2,9%** | **2,7%** | **13,3%** |  |
