# Supplementary material for: Genetic diversity and structuring across the range of a widely distributed ladybird: focus on rear‐edge populations phenotypically divergent
Source: Ecol Evol. 2016 Jul 13;6(15):5517–29. doi: 10.1002/ece3.2288 (PMC4984522; doi:10.1002/ece3.2288)
Supplement: Supplementary file 6 — Table S3. Genetic signatures of demographic changes in Coccinella septempunctata identified with BOTTLENECK, significant deficiency of heterozygotes are indicated as “expansion” and excess as “bottleneck”. [file ECE3-6-5517-s006.docx]

Genetic diversity and structuring across the range of a widely distributed ladybird:
focus on rear-edge populations phenotypically divergent

**Table S3**. Genetic signatures of demographic changes in *Coccinella septempunctata* identified with BOTTLENECK, significant deficiency of heterozygotes are indicated as “expansion” and excess as “bottleneck”.

| **Population** | **All 18 loci** | | **13 loci (NA freq. <15%)** | | **6 loci (NA freq. <3%)** | |
| --- | --- | --- | --- | --- | --- | --- |
| **Algeria, Alger** | **expansion** | ***** SMM** | **expansion** | *** SMM** | **expansion** | **** SMM** |
| **Algeria, Biskra** | **expansion** | ***** SMM** | **expansion** | *** SMM** | **expansion** | *** SMM** |
| Belgium, Gembloux | expansion | *** SMM | expansion | *** SMM | expansion | * SMM, * TPM |
| **China, Chengdu** | **bottleneck** | **** TPM** | **expansion** | *** SMM** | no signal |  |
| Czech Republic, Prague | expansion | *** SMM | expansion | *** SMM | expansion | *** SMM, * TPM |
| Denmark, Skagen | expansion | *** SMM | expansion | *** SMM, * TPM | expansion | * SMM, * TPM |
| France, Toulouse | expansion | *** SMM | expansion | *** SMM | expansion | * SMM |
| India, Lucknow city | expansion | * SMM | expansion | * SMM | expansion | * SMM, * TPM |
| India, Shimla | expansion | *** SMM | expansion | *** SMM | expansion | * SMM |
| Iran, Saveh | expansion | *** SMM | expansion | *** SMM | expansion | * SMM |
| Italy, Perugia | expansion | *** SMM | expansion | * SMM | expansion | * SMM |
| **Japan, Tsuruoka** | **bottleneck** | *** TPM** | **bottleneck** | *** TPM** | no signal |  |
| Poland, Tomianski | expansion | *** SMM | expansion | *** SMM | expansion | ** SMM |
| Portugal, Lisbon | expansion | *** SMM | expansion | *** SMM | expansion | ** SMM |
| Spain, Victoria-Gasteiz | no signal |  | expansion | * SMM | expansion | * SMM |
| Sweden, Alnarp | expansion | *** SMM | expansion | *** SMM | expansion | * SMM |
| Switzerland, Delemont | expansion | *** SMM | expansion | *** SMM, * TPM | expansion | * SMM |
| United Kingdom, Norwich | expansion | *** SMM | expansion | *** SMM | expansion | ** SMM, * TPM |

Analyses were carried using one-tailed Wilcoxon’s test under the stepwise mutation model (SMM) and the two-phased mutation model (TPM). Significant values are indicated: * for p<0.05, ** P<0.01, *** p<0.001. The populations from Algeria, China and Japan are in bold.
